# A method to quantitatively characterize the formation and dissociation of tumor cell clusters using light transmission aggregometry

**DOI:** 10.1002/1878-0261.13723

**Published:** 2024-09-05

**Authors:** Regina Komal Kottana, Brian Schnoor, Anne‐Laure Papa

**Affiliations:** ^1^ Department of Biomedical Engineering, School of Engineering and Applied Science The George Washington University Washington DC USA

**Keywords:** alteplase, cancer cell clusters, fibrinolytic agents, light transmission aggregometry, platelet–tumor cell aggregates, tenecteplase

## Abstract

In this paper, we have modified the workflow of the traditional light transmission aggregometry (LTA) protocol to characterize tumor cell clusters *in vitro* in a quantifiable and multifaceted manner. Circulating tumor cell (CTC) clusters have high metastatic potential compared to single tumor cells traveling in the bloodstream. Thus, engineering new therapeutic strategies that specifically target this CTC population is essential. To accomplish this, quantifiable methods to characterize their therapeutic effect on tumor cell clusters is a prerequisite. The method presented here enables the user to precisely quantify the dissociation of cancer cell clusters in the presence of clinically relevant fibrinolytic agents, such as alteplase and tenecteplase. The efficacy of the fibrinolytic agents can be quantified using this *in vitro* assay, prior to conducting preclinical studies. Here, we have obtained the fibrinolytic activity data in terms of lag time to the initiation of tumor cell dissociation, time to 25% dissociation, and trend of dissociation over time. To validate the assay, cell counts and phase‐contrast microscopy images were recorded over time. Further, we explored an LTA‐assisted preparation of platelet–tumor‐cell clusters of calibrated size for potential downstream testing/applications. To assess whether the assay is applicable to characterize the dissociation of cancer cell clusters in the presence of platelets, we added low (50 000 platelets·μL^−1^), normal (200 000 platelets·μL^−1^) and high (450 000 platelets·μL^−1^) concentrations of platelets to the tumor cell clusters. In addition to dissociation parameters, microcopy images were recorded over time to validate the assay and enabled the enumeration of clusters and single cells. The correlative light electron microscopy (CLEM) technique was utilized to visualize the morphology and composition of platelet–tumor cell clusters.

## Graphical abstract

A method was developed to quantifiably characterize the dissociation of tumor cell clusters *in vitro* by modifying a traditional light transmission aggregometry protocol. The efficacy of this method was demonstrated by characterizing tumor cell cluster dissociation by fibrinolytic agents. Further, this method was developed to produce consistent platelet–tumor cell clusters for downstream applications.

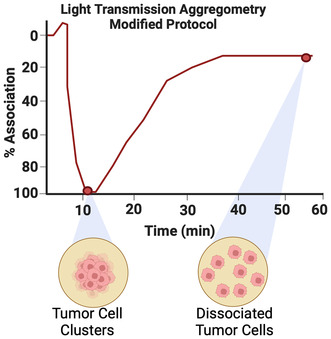

**Abbreviations**
CLEM: correlative light electron microscopy
CTC: circulating tumor cell
DMEM: Dulbecco's Modified Eagle's Medium
FBS: fetal bovine serum
HMDS: hexamethyldisilane
HyD: hybrid detectors
LTA: light transmission aggregometry
PC: platelet concentrate
PMMA: polymethyl methacrylate
PMT: photomultiplier tube
PPP: platelet‐poor plasma
PRP: platelet‐rich plasma
SEM: scanning electron microscopy
TNK: tenecteplase
tPA: tissue plasminogen activator
uPA: urokinase‐type plasminogen activator
WBC: white blood cells

## Introduction

Cancer dissemination and metastasis is enabled by the ability of cancer cells to escape the primary tumor, intravasate in the bloodstream and subsequently extravasate from a blood vessel and seed distant tissues [1]. During this process, circulating tumor cells (CTC) travel either as single cells or as a group of multiple cells, also called CTC clusters or CTC microemboli [2]. CTC clusters navigating the bloodstream have a greater metastatic potential than single circulating tumor cells [3] as traveling in a group confers them with enhanced survival advantage [4, 5]. Indeed, tumor cells cushion each other while navigating the bloodstream, resulting in a greater survival while they undergo shear stress [2]. CTC clusters are often detected in early‐stage breast cancer [6], as well as in metastatic epithelial cancers [3]. In addition, their detection in metastatic patients is associated with poor prognosis [3, 7]. CTC clusters can derive from cohesive groups of cells that enter into the blood as a cluster [3], or in some cases, from cancer cells that aggregate in the bloodstream [8]. Prior work introducing cancer cells into *in vivo* models has shown that tumor cells form clusters with a network of fibrin and platelets [9, 10, 11]. Furthermore, when CTC clusters are detected in *ex vivo* patient blood samples, the tumor cells are typically found associated with platelets in this manner [12, 13]. Current research emphasizes the detection of such cancer cell microemboli in liquid biopsies (i.e. blood specimen of cancer patients) using either CellSearch®, microfluidic chips or filtration devices [4], so as to leverage them as patient monitoring tools. Additionally, intravascular targeting of CTC clusters could represent a future strategy to address this highly metastatic subpopulation. For instance, urokinase‐type plasminogen activator (uPA), an approved fibrinolytic drug, has demonstrated a significant efficiency in decreasing the metastatic potential of CTC clusters in a murine breast cancer model [14]. As more strategies are developed to target and eliminate CTC clusters [14, 15, 16], there is a critical and unmet need in developing quantitative methods for cluster analysis in the context of therapeutic development. This would include tools that supports the quantification of efficiency of treatments in human plasma specimen spiked with tumor cell lines. In order to address this need, we introduce the use of a Light Transmission Aggregometry (LTA) as a tool to study tumor cell cluster *in vitro*. Additionally, we have also explored the use of this method to monitor the production of platelet–tumor cell clusters for downstream applications. Prior work has created tumor cell clusters to investigate their increased metastatic potential [17, 18]. However, these studies have not created clusters incorporating platelets or fibrin as is found *in vivo*. Furthermore, additional work has explored the use of modified platelets or platelet membrane functionalized particles to target CTCs in circulation [19, 20], further reinforcing the need for a method to monitor and generate platelet–tumor cell clusters. Our developed method enables, for the first time, the preparation of tumor cell clusters incorporating platelets that can then be assessed in subsequent experiments.

LTA is a quantitative method used to analyze platelet aggregation [21]. The concept of LTA was independently described by Born [22] and O'Brien [23] in the 1960's and it has ever since been considered a gold standard for platelet aggregation testing, to identify or diagnose platelet function disorders [24]. LTA has also been used in research settings to study tumor cell‐induced platelet aggregation [25]. However, this technique has not been utilized to study the tumor cell clusters themselves. Therefore, we repurposed the LTA to study the effect of acute therapeutics such as fibrinolytics on tumor cell dissociation in a quantitative and reproducible way. We also introduce LTA as a way to monitor the production of platelet–cancer cell clusters.

We used human plasma samples spiked with breast (MDA‐MB‐231) and lung (A549) cancer cell lines in the presence of calcium chloride to initiate the formation of a fibrin‐rich tumor cell cluster prior to treating samples with two FDA‐approved fibrinolytic drugs, Alteplase (tPA) and Tenecteplase (TNK). Fibrinolytic drugs enable the conversion of plasma plasminogen into plasmin, that itself cleaves fibrin network in fibrin degradation products. The fibrinolytic dissociation was quantified by the lag time to the initiation of tumor cell dissociation, time to 25% dissociation and trend of dissociation over time. This method was also validated using both microscopy and changes in cell counts measured from sample supernatants overtime. Thus, this method using the LTA can be used to reliably characterize the dissociation of tumor cell aggregate/clusters *in vitro* in a quantifiable manner. Further, we have also successfully produced platelet–tumor cell clusters for use in downstream applications.

## Materials and methods

### Materials

Clinical grade tPA (alteplase) and TNK (tenecteplase) were obtained through a materials transfer agreement with Genentech (San Francisco, CA, USA). The calcium chloride (CaCl_2_, cat# C1016‐100G), sodium chloride (NaCl, cat# S7653‐250G), sodium bicarbonate (NaHCO_3_, cat# S5761‐500G), potassium chloride (KCl, cat# P9333‐500G), disodium phosphate (Na_2_H_2_PO_4_, cat# S7907‐100G), magnesium chloride (MgCl_2_, cat# M8266‐100G), HEPES (cat# 83537‐100ML), and phosphate‐buffered saline (PBS, cat# P4417‐100TAB), trypan blue (cat# T8154‐20ML) and Collagen G (cat# C8919‐20ML) were obtained from Sigma‐Aldrich, (St. Louis, MO, USA). Control plasma (cat# 30‐201) was obtained from R2 diagnostics (South Bend, IN, USA). Alprostadil (PGE1, cat# 1620) was obtained from Tocris Bioscience (Minneapolis, MN, USA). LTA cuvettes (cat# P/N 312), disposable stir bars (cat# P/N 311), 0.1–10 μL tips (cat# P/N 334), and 10–100 μL tips (cat# P/N 337) were purchased form Chrono‐Log (Havertown, PA, USA). EasySep Direct Human PBMC Isolation Kit (cat# 19654) was obtained from STEMCELL Technologies (Cambridge, MA, USA). Purified anti‐human CD41 (cat# 303702) and purified anti‐mouse/human CD45R/B220 (cat# 103201) were obtained from Biolegend (San Diego, CA, USA). Hoechst 33342, trihydrochloride, trihydrate (cat# H3570), Cellmask Deep Red Plasma Membrane Stain (cat# C10046), goat anti‐Mouse IgG (H+L) secondary antibody, Alexa Fluor 488 (cat# A11029), goat anti‐rat IgG (H+L) secondary antibody, Alexa Fluor 546 (cat# A11081), and ProLong glass antifade mountant (cat# P36980) was obtained from Invitrogen (Waltham, MA, USA). Improved Neubauer hemocytometers (cat# CD‐NEUI‐0025) were obtained from Bioanalytic GmbH (Umkirch, Germany). Healthy human whole blood with ACD‐A anticoagulant was commercially sourced from BioIVT (Westbury, NY, USA), under the approval from the Institutional Biosafety Committee at the George Washington University (protocol # IBC‐19‐007).

### Cell culture

MDA‐MB‐231 (RRID:CVCL_0062) breast cancer cells (cat# HTB‐26) and A549 (RRID:CVCL_0023) lung cancer cells (cat# CCL‐185) were obtained from ATCC. The cell lines were authenticated using ATCC's Human cell STR profiling service and the experiments were performed with mycoplasma‐free cells. These cells were cultured in 75 cm^2^ Falcon tissue culture flasks in Dulbecco's Modified Eagle's Medium (DMEM) with 4.5 g·L^−1^ D‐glucose, L‐glutamine, 110 mg·L^−1^ sodium pyruvate (Gibco, Waltham, MA, USA, cat# 11995‐065) supplemented with 10% Fetal Bovine Serum (FBS, Gibco, cat# 26140‐079) and 1% Pen Strep (penicillin streptomycin, Gibco, cat# 15140‐122). The cells from either cell lines were collected using 0.25% Trypsin – EDTA (Gibco, cat# 25200‐056) and resuspended in Tyrode buffer (136 mm NaCl, 12 mm NaHCO_3_, 2.9 mm KCl, 0.34 mm Na_2_H_2_PO_4_, 1 mm MgCl_2_, and 10 mm HEPES) after neutralizing the trypsin. The cell concentration was determined with a LUNA‐II™ cell counter (Logos Biosystems Inc., Gyeonggi‐do, South Korea) and a trypan blue viability stain.

MDA‐MB‐231 cells were obtained using the cell‐scrapping method for the production of the platelet–tumor cell clusters, as trypsin would significantly impact integrins involved in their interaction. For fluorescent imaging performed to validate the platelet–cancer cell cluster association/dissociation, the cancer cells were further stained with 10000× diluted Hoechst 33342 nucleic acid stain for 5 min at 37 °C.

Similarly, the MDA‐MB‐231 cells used during the production of platelet–tumor cell clusters were stained with 10,000× diluted Hoechst 33342 nucleic acid stain for 5 min and the 1000× diluted Deep Red Plasma Membrane stain for 15 min at 37 °C.

### Light transmission aggregometry (LTA) for cancer cell cluster association/dissociation

A new method leveraging light transmission aggregometry was developed to study tumor cell association and dissociation using a model 490 4 + 4 aggregometer (Chronolog Corporation, Havertown, PA, USA). This LTA protocol was designed to promote tumor cell clustering and to monitor association/dissociation of the tumor cells. The quantitative characterization of the association/dissociation of the tumor cell clusters was achieved by replacing the platelet‐poor plasma (PPP) reference used in traditional LTA by an optical reference sample prepared as described below.

#### Aggregometry optical reference preparation

(1) Control Plasma made of a 15 healthy donor pool (R2 Diagnostics, cat# 30‐201) was warmed at 37 °C for 10 min. (2) LTA cuvettes were preloaded with a stir bar, as well as 20 μL of 62.5 mm CaCl_2_. The stirring speed was set to 1200 rpm. (3) A cuvette with Tyrode solution alone is placed in the reference well of the device, in order to observe the association of the tumor cell clusters during the production of the optical reference solution. (4) The tumor cell suspensions were diluted to 4000 cells·μL^−1^ in Tyrode buffer and were mixed with the warmed plasma in a 1 : 1 volume ratio. (5) 250 μL of the plasma‐tumor cell mixture was then added to the LTA cuvette in the test well. (6) After 10 min, the supernatant was collected and subsequently filtered through a 0.22 μm filter (Thermofisher, Waltham, MA, USA, cat# F2500‐16) to represent the 100% transmittance reference for the associated samples. (7) The prepared reference solution was then loaded in the reference holder. Separate references were used for respective cell lines and cell concentrations where applicable.

#### Association of tumor cell clusters

The above‐mentioned steps (1, 2) were repeated. (8) The appropriate optical reference created using steps (1–7) was inserted into the reference well and steps (4, 5) were repeated with the new optical reference. (9) The trace of the sample was then immediately set to baseline (see 4 + 4 model user manual) [26] prior to recording the stable clustering of tumor cells with fibrin. Using the above‐mentioned protocol leads to the formation of a large fibrin‐rich cancer cell aggregate/cluster at 10 min.

#### Dissociation of tumor cells

Clinical grade tissue plasminogen activator TNK and tPA (Genentech, San Francisco, CA, USA) at concentrations of 0.25, 0.5, and 1 μg·mL^−1^ were used as fibrinolytic agents to break down the fibrin and release the cancer cells. (10) The fibrinolytic agents were added at the 10 min time point. The dissociation with the fibrinolytic agents was measured over 50 min. TNK and tPA were freshly thawed and kept on ice throughout the experiment.

### Light transmission aggregometry (LTA) for platelet–cancer cell cluster association/dissociation

To include platelets in the clusters, platelet concentrate (PC) was made by isolating platelet‐rich plasma (PRP) from ACD‐A human whole blood via centrifugation at 150 ***g*** for 20 min. PRP was then incubated with 140 nm prostaglandin E1 (PGE1, Tocris, cat# 1620) for 10 min and subsequently centrifuged at 1000 ***g*** for 20 min. The obtained platelet pellet was then resuspended in 250 μL of Tyrode buffer leading to a platelet concentrate (PC) suspension. The cancer cells were stained with Hoechst 33342 to enable fluorescence imaging. The steps (1–10) from “Light transmission aggregometry (LTA) for cancer cell cluster association/dissociation” were repeated with a modification to step (4). Step (4) was modified by adding calculated volumes of PC to the warmed plasma to account for the low (50 000 platelets·μL^−1^), normal (200 000 platelets·μL^−1^) and high (450 000 platelets·μL^−1^) concentration of platelets, before further mixing the cancer cells (4000 cells·μL^−1^ in Tyrode buffer) in a 1 : 1 volume ratio with a final volume of 250 μL.

### Formation and monitoring of platelet–tumor cell clusters

We followed the method described in the above LTA method section with the following alterations: the amount of plasma used was reduced to 5% of the total volume to reduce fibrinogen concentration and thus produce smaller fibrin‐bound platelet–tumor cell clusters. Additionally, the concentration of CaCl_2_ used was reduced to 1 mm. Finally, two cell concentrations (3000 and 1500 cells·μL^−1^) were chosen to form these clusters based on preliminary data.

MDA‐MB‐231 cells were stained with Hoechst 33342 and deep red plasma membrane stain to allow for fluorescent imaging and Correlative Light Electron Microscopy (CLEM) of the clusters formed. The reference was prepared as described in the previous LTA section, though the platelets were not included in the preparation.

First, 24 μL of 12.5 mm CaCl_2_ and a stir bar were preloaded in a cuvette and placed in the test well holders. In a separate cuvette, PC (200 000 platelets·μL^−1^) was then added to the 5% plasma and Tyrode buffer and warmed at 37 °C for 10 min. Tumor cells (3000 or 1500 cells·μL^−1^) were subsequently added to the warmed suspension for a combined volume of 276 μL. This sample was then transferred to the cuvette containing the 12.5 mm CaCl_2_ solution for a final volume of 300 μL. The LTA channel was immediately baselined, and the measurement was started. 5 μL supernatant samples were taken at 10, 60, and 120 min to image cancer cell clusters in suspension.

### Formation of platelet–tumor cell clusters in whole blood

The steps from “Formation and monitoring of platelet–tumor cell clusters” were repeated, the 5% PPP used was replaced with 5% whole blood. The platelet concentration was adjusted to 200 000 platelets·μL^−1^ by adding appropriate volume of PC. Instead of using the LTA to characterize the formation of clusters, 5 μL supernatant samples were taken at 10, 60, and 120 min and imaged to monitor the formation of cancer cell clusters in suspension.

### Enumeration of cells in supernatants

For the LTA cancer cell cluster association/dissociation protocol, the cells were enumerated using the LUNA‐II cell counter. A 5 μL sample of the supernatant was collected from the top of the cuvettes at 0, 10, 35, and 60 min time points and were mixed 1 : 1 v : v with trypan blue. For the formation and monitoring of the platelet–cancer cell clusters protocol 5 μL samples of the supernatant collected at the 10, 60, and 120 min timepoints were 6x diluted and transferred to the improved Neubauer hemocytometer. The clusters and cells were counted manually.

### Microscopy

An Olympus CKX53 microscope (Olympus, Tokyo, Japan) with a RETIGA R1 (Q Imaging, Tucson, AZ, USA) camera was used for imaging the samples. For the “LTA cell cluster association/dissociation” method, the microscopy images were taken by collecting 5 μL of the supernatant from the surface of the cuvettes and then 4× diluted with PBS in a 384 well plate (Thermofisher, cat# 142761). The 384 well plate was then imaged using a 10× (Olympus, cat# N5229200) objective lens. The supernatant sampling and imaging were repeated for each time point. For the “Formation and monitoring of platelet–tumor cell clusters” protocol, 5 μL of the collected supernatant was added to the improved Neubauer hemocytometer cell counting slides and a 10× (Olympus, cat# N5229200) objective lens was used for magnification. The supernatant sampling and imaging were repeated for each time point.

### Correlative light electron microscopy (CLEM) for the visualization of platelet–tumor cell clusters

First, grids were printed on the Si wafers to easily align/correlate the confocal microscopy images with the scanning electron microscopy (SEM) images. The sample grids were prepared by coating a Si wafer with 600 nm polymethyl methacrylate (PMMA), and then patterning it using electron beam lithography (Raith Voyager, 50KV, DE). After development in MIBK : IPA 1 : 3 for 60 s, wafers were cleaned in a plasma asher (30 s at 30 W) and finally etched by the Bosch process (Plasmatherm Versaline) for 15 loops. The PMMA was then removed by acetone, leaving the grid pattern etched into the Si. All Si wafers were cleaned with ethanol and DI water, then coated with 0.1 mg·mL^−1^ collagen G and incubated for 1 h. The “Formation and monitoring of platelet–tumor cell clusters” protocol was performed with either 5% PPP or 5% whole blood, and 50 μL samples were collected at the 120 min time point. The collected samples were then transferred to the collagen G‐coated silicon wafers in a 24‐well plate and allowed to settle for 30 min. 4% paraformaldehyde was added and incubated for 20 min. The 24‐well plate with the samples was then centrifuged at 150 ***g*** for 5 min, and the 4% paraformaldehyde was replaced with fresh 4% paraformaldehyde and allowed to incubate overnight. The samples were then rinsed 4 times with PBS and incubated with purified anti‐human CD41 and purified anti‐mouse/human CD45R/B220 primary antibodies at a dilution of 1 : 100 for 2 h at room temperature. They were then stained with the goat anti‐mouse IgG (H + L) secondary antibody, Alexa Fluor™ 488, and goat anti‐rat IgG (H + L) secondary antibody, Alexa Fluor™ 546 secondary antibodies at a dilution of 1 : 300 for 1 h at room temperature in the dark. Imaging was then performed using the Leica TCS SP8 MP (Leica, Teaneck, NJ, USA) confocal microscope. The white laser was set to 70% power, and a combination of photomultiplier tube (PMT) and hybrid detectors (HyD) was used. The grids were imaged using reflection microscopy. The 40x water deep immersion objective (HC PL APO 40x/1.10 W CORR CS2/Leica) was used for z‐stack imaging. The white blood cells (WBC) control samples were prepared by separating the WBC from whole blood using the EasySep Direct Human PBMC Isolation Kit. After Isolation, the WBCs were stained with the primary and secondary antibodies mentioned above and fixed with 1% paraformaldehyde. The stained WBC sample was smeared on a glass slide and covered with high precision No. 1.5H coverslip using the ProLong glass antifade mounting media. A 63× objective (HC PL APO 63×/1.40 Oil CS2) was used to image the WBC cells. The full z‐stack images obtained were edited using the imaris 10.1 (Bitplane) software to improve visualization in 3D mode. All samples in coated silicon wafers were then processed for SEM imaging. The samples were further fixed with 2.5% glutaraldehyde, 1% paraformaldehyde, and 0.12 m sodium cacodylate buffer, pH 7.4 at 4 °C. Buffer rinses with 0.12 m sodium cacodylate buffer, pH 7.4 were performed 3 times for 10 min each time. Post‐fixation was performed using 1% osmium tetroxide and 0.12 m sodium cacodylate buffer, pH 7.4 in the dark for 60 min. After post‐fixation, the samples were rinsed 4 times with DI water for 10 min each time under shaking. The samples were then dehydrated by sequentially adding 15%, 35%, 50%, 70%, 80%, and 95% ethanol and incubating for 10 min each time. 100% ethanol was then added 3 times and incubated for 10 min each time. After dehydration, the samples were placed in hexamethyldisilane (HMDS) for 15 min. Fresh HMDS was replaced, and the samples were allowed to evaporate overnight. No sputter coating was performed. For scanning electron microscopy, the FEI Teneo LV FEG SEM was used. SEM imaging was performed at high vacuum, in OptiPlan mode (high‐resolution) using low currents (1 kV, 50pA beam current), 5 us dwell time, using the Everhart–Thornley detector (ETD) detector (secondary electrons), at 6.5 mm working distance and 10 000x magnification. The pixel size was 13.48 nm. Maps 3.22 software was used for the tile and stitching of the image obtained.

### Data processing

The raw data obtained from the aggrolink opti8 software (Chrono‐Log) for tumor cell association/dissociation of MDA‐MB‐231 (Fig. S1A) and A549 (Fig. S1C) tumor cell clusters were smoothed in graphpad prism 9 in order to reduce noise by using a second order polynomial with 10 neighbors prior to calculating specified characteristics (Fig. S1B,D). All subsequently measured characteristics: lag time, time to 25% dissociation, and dissociation over time, were determined using the data obtained after the addition of the fibrinolytic agent at the 10 min time point.

### Delay of clot formation

In order to assess the comparative effectiveness of tPA and TNK a delay of clot formation absorbance assay was used. In each well of a 96‐well plate, 80 μL of control plasma from R2 diagnostics was added along with treatments. Clot formation was initiated by adding thrombin to each well.

The treatment conditions were 1, 0.5, and 0.25 μg·mL^−1^ final concentrations of tPA and TNK respectively. For each treatment condition, 10 μL of the treatment solution was added to their respective wells. Then clot formation was induced by adding 10 μL of 10 U·mL^−1^ thrombin solution to each well. The absorbance was then measured at 405 nm using a Spectramax ID5 plate reader every minute for 1 h with 15 s of linear shaking between measurements. After the curves were obtained, the change in absorbance from the initial measurement was determined.

A positive control was prepared by adding 10 μL of saline to the 80 μL of plasma before inducing clot formation with thrombin and measuring as above. Negative control conditions were prepared for each treatment condition using the same concentrations described above, but without adding the thrombin solution. Instead, 10 μL of saline was added instead to maintain the same optical conditions without inducing clot formation. A final negative control with only 80 μL of plasma and 20 μL of saline was also measured.

### Statistical analysis

A one‐way ANOVA analysis with Tukey's multiple comparison test was used to analyze the lag time and time to 25% dissociation characteristics for the tumor cell dissociation curves with and without platelets. A two‐way ANOVA analysis with a Sidak's multiple comparison test was used to analyze the fibrinolytic dissociation of the tumor cell clusters and the supernatant cell counts over time within a treatment group, while a Tukey's multiple comparison test was used to compare the fibrinolytic dissociation of tumor cells and the supernatant cell counts between groups at the 60 min time point. A two‐way ANOVA analysis with a Sidak's multiple comparison test was used to determine the effect of cell concentration on platelet–tumor cell cluster aggregation at each time point, while a two‐way ANOVA analysis with a Tukey's multiple comparison test was used to analyze the measured platelet–tumor cell cluster formation over time within each concentration condition. Two‐way ANOVA analyses with Tukey's multiple comparison tests were also used to compare the supernatant cancer cell cluster or single cell concentration over time within condition groups, between samples with different concentration conditions at each time point, and between samples in PPP and whole blood for each concentration and time point. A two‐way ANOVA analysis with Tukey's multiple comparison correction was also performed to compare the absorbance change due to clot formation for each tPA or TNK treatment or control group. Finally, two‐way ANOVA analyses with Sidak's multiple comparison tests were conducted to compare the fibrinolytic dissociation of tumor cell clusters with the same treatment and at the same time point across different platelet conditions (Tables S1 and S2).

## Results

### Tumor cell dissociation using fibrinolytic drugs

**Fig. 1:**
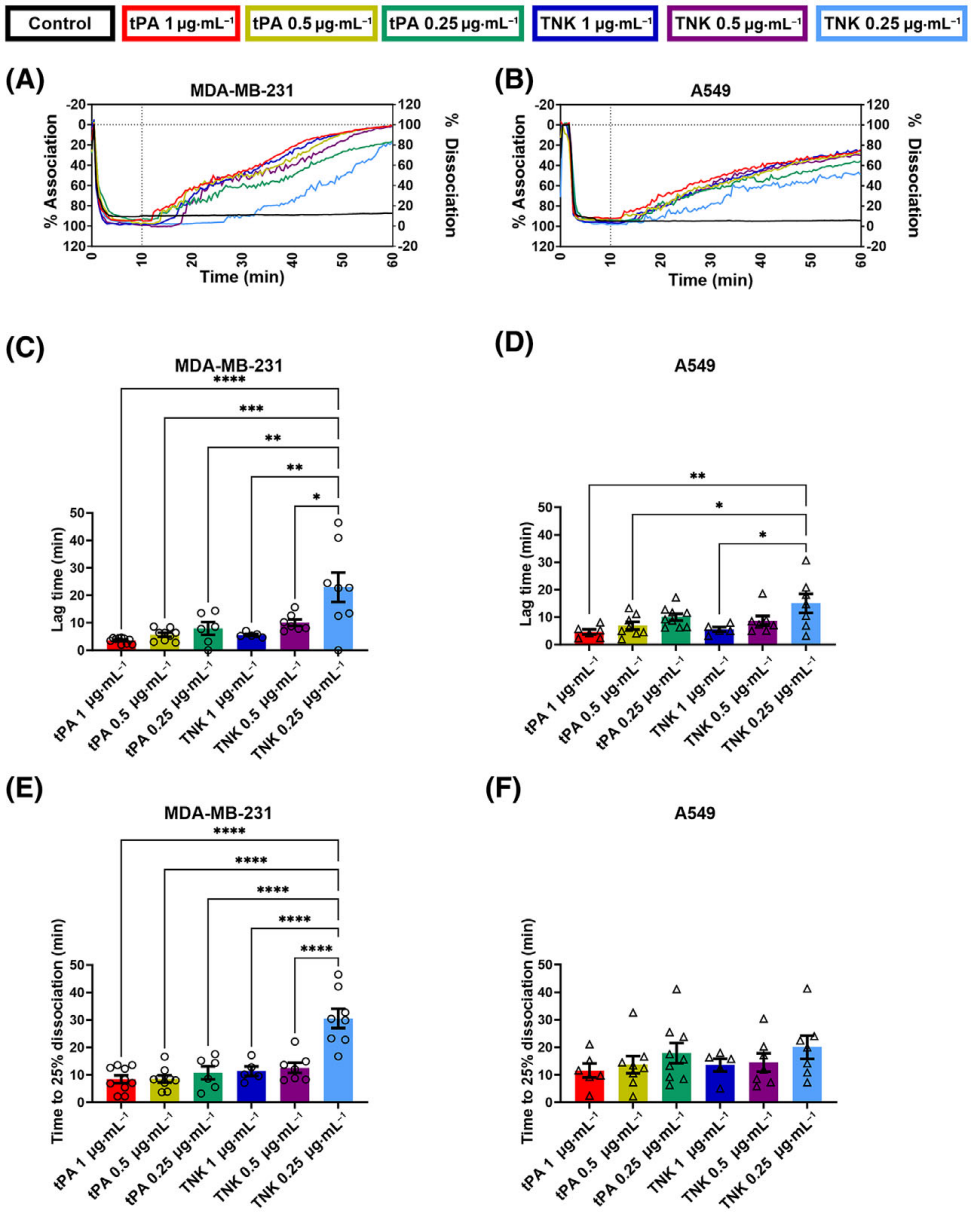
Characterization of light transmission aggregometry (LTA) curves for the association and dissociation of MDA‐MB‐231 and A549 cancer cells. The LTA curves for the MDA‐MB‐231 and the A549 tumor cell association, and dissociation after treatment with different concentrations of tissue plasminogen activator (tPA) and tenecteplase (TNK), were analyzed to produce an average curve (A, B), determine the dissociation lag time (C, D), and measure the time to 25% disassociation (E, F). For each condition, the treatment is added at 10 min as indicated with a dotted line on the average curve (A, B). The lag time (C, D) and time to 25% dissociation (E, F) are measured from the addition of the treatment. These conditions included a control (MDA‐MB‐231: *n* = 6, A549: *n* = 7), 1 μg·mL^−1^ tPA (MDA‐MB‐231: *n* = 10, A549: *n* = 6), 0.5 μg·mL^−1^ tPA (MDA‐MB‐231: *n* = 9, A549: *n* = 8), 0.25 μg·mL^−1^ tPA (MDA‐MB‐231: *n* = 6, A549: *n* = 9), 1 μg·mL^−1^ TNK (MDA‐MB‐231: *n* = 5, A549: *n* = 5), 0.5 μg·mL^−1^ TNK (MDA‐MB‐231: *n* = 7, A549: *n* = 7), and 0.25 μg·mL^−1^ TNK (MDA‐MB‐231: *n* = 8, A549: *n* = 7). To determine the statistical significance of differences between conditions, a one‐way ANOVA analysis with Tukey's multiple comparison test was performed (*P ≤ 0.05, **P ≤ 0.01, ***P ≤ 0.001, ****P ≤ 0.0001). For all conditions, the mean and standard error of the mean (SEM) are displayed.

The association of the MDA‐MB‐231 and A549 cancer cells (0–10 min) was consistent with an average percent association of 95.8 ± 7.6% (*n* = 52) for all MDA‐MB‐231 samples and 94.9 ± 7.3% (*n* = 49) for all A549 samples (Fig. 1). Additionally, the control group demonstrated that this association was firm for at least 1 h without a fibrinolytic treatment for both cell lines (Fig. 1A,B, black traces). When the fibrinolytic agent was added at 10 min in the other conditions, all of them showed a strong dissociation with some variations in the time before the start of dissociation. In particular, the lowest TNK treatment concentration of 0.25 μg·mL^−1^ had a much less steep curve, began dissociating later, and did not completely disassociate the clustered MDA‐MB‐231 or A549 over the course of 1 h (Fig. 1A,B).

It is also interesting to note that there was a distinct difference between the dissociation curves for the MDA‐MB‐231 and A549 cell lines (Fig. 1A,B). The average dissociation curve for each treatment group with MDA‐MB‐231 cells was markedly steeper than the corresponding curves for the A549 cancer cells. Correspondingly, the percentage of dissociation at 60 min (from maximum aggregation at 10 min) for the MDA‐MB‐231 was markedly higher than the corresponding dissociation of A549 (e.g. 92.3% versus 67.0% when cells were treated with 1 μg·mL^−1^ tPA, Fig. 1A,B). This trend of lower dissociation at 60 min with A549 holds true for all treatment groups.

The lag time between the introduction of the fibrinolytic agent and the beginning of the dissociation (set at 5% dissociation detected) was greater for 0.25 μg·mL^−1^ TNK compared to 1 μg·mL^−1^ TNK (*P* = 0.0011 for MDA‐MB‐231 and *P* = 0.0290 for A549), as well as for the 0.25 μg·mL^−1^ TNK as compared to the 0.5 μg·mL^−1^ TNK (*P* = 0.0107 for MDA‐MB‐231) (Fig. 1C,D). Furthermore, there was a non‐significant but consistent trend of increasing lag times for lower concentrations of fibrinolytic treatment for both cell lines as well (Fig. 1C,D). Finally, the associated MDA‐MB‐231 cells treated with tPA at the 0.25 μg·mL^−1^ concentration had a significantly lower lag time than those treated with the same concentration of TNK (*P* = 0.0034) (Fig. 1C).

Regarding the time to 25% dissociation for MDA‐MB‐231 clusters (Fig. 1E), the 0.25 μg·mL^−1^ TNK treatment condition had a significantly longer time to 25% dissociation compared to the 1 μg·mL^−1^ TNK (*P* < 0.0001) or even the 0.5 μg·mL^−1^ condition (*P* < 0.0001), while tPA did not show significant differences among groups within this range of concentrations (0.25–1 μg·mL^−1^). Additionally, the 0.25 μg·mL^−1^ TNK treatment group also required significantly longer to reach 25% dissociation for MDA‐MB‐231 cells compared to the 0.25 μg·mL^−1^ tPA group (*P* < 0.0001), which is consistent with the lag time analysis (Fig. 1E). Although non‐significant, a trend towards longer dissociation times with lower concentrations of fibrinolytic treatments was seen with A549 cells (Fig. 1F). Our rationale for choosing 25% dissociation was that this measurement best highlights differences among groups in our experimental conditions. In addition, fibrinolytics are potent drugs that display rapid fibrinolysis; thus we focused on early dissociation. However, it is a reasonable assumption that different or multiple percentages of cancer cell dissociation could be envisioned to be reported with this method depending on the drug and the cells used.

### Validation of LTA as a method for measuring tumor cell cluster dissociation

**Fig. 2:**
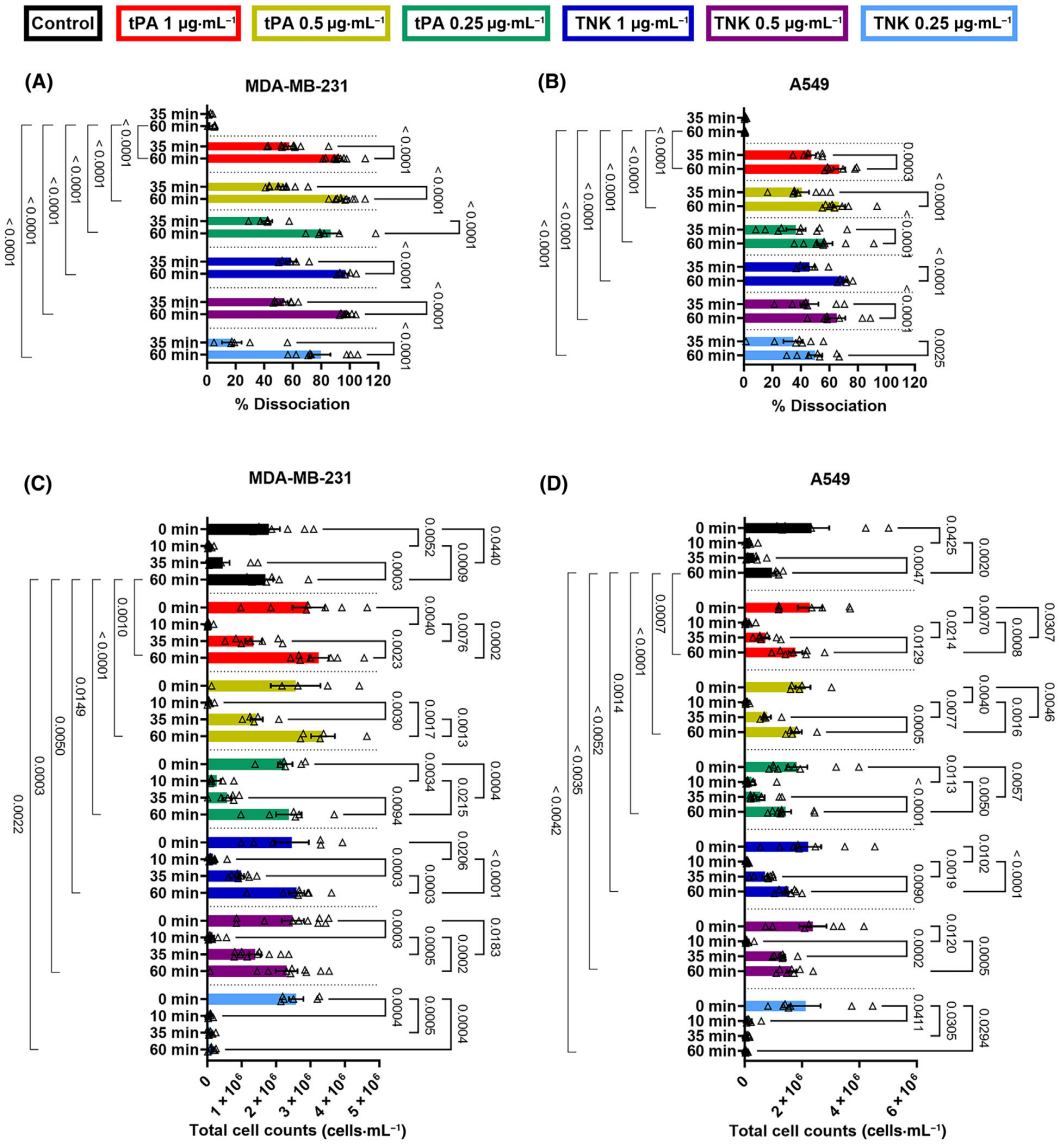
Validation of light transmission aggregometry (LTA) analysis via cell count measurement of MDA‐MD‐231 and A549 cancer cells. LTA analysis of the dissociation of (A) MDA‐MB‐231 and (B) A549 with various tissue plasminogen activator (tPA) and tenecteplase (TNK) conditions at 35 and 60 min. For validation, the number of (C) MDA‐MB‐231 and (D) A549 in the suspension were counted at 0 (when cancer cells were introduced), 10, 35, and 60 min. The tPA and TNK conditions were: control (MDA‐MB‐231: *n* = 6, A549: *n* = 7), 1 μg·mL^−1^ tPA (MDA‐MB‐231: *n* = 10, A549: *n* = 6), 0.5 μg·mL^−1^ tPA (MDA‐MB‐231: *n* = 9, A549: *n* = 8), 0.25 μg·mL^−1^ tPA (MDA‐MB‐231: *n* = 6, A549: *n* = 9), 1 μg·mL^−1^ TNK (MDA‐MB‐231: *n* = 5, A549: *n* = 5), 0.5 μg·mL^−1^ TNK (MDA‐MB‐231: *n* = 7, A549: *n* = 7), and 0.25 μg·mL^−1^ TNK (MDA‐MB‐231: *n* = 8, A549: *n* = 7). A two‐way ANOVA analysis with a Sidak's multiple comparison test was used to analyze the fibrinolytic dissociation of the tumor cell clusters and the supernatant cell counts over time within a treatment group, and a Tukey's multiple comparison test was used to compare the fibrinolytic dissociation of tumor cells and the supernatant cell counts between groups at the 60 min time point. For all conditions, the mean and standard error of the mean (SEM) are displayed.

Next, we quantified the dissociation seen by LTA at various time points (Fig. 2A,B) and compared the data to the cancer cell count in the same sample supernatant over time (i.e. direct enumeration of cells that are not trapped in the fibrin clot) (Fig. 2C,D). This cell count measurement provided a basis to validate the dissociation percentages seen by LTA at 10, 35 and 60 min. We observed a similar trend for both tPA and TNK at different concentrations. The percentage of dissociation of tumor cells from the fibrin clot over time is dependent on the concentrations of the fibrinolytic agent. We consistently observed the association of tumor cells at 10 min, with sustained retention in the clot if no fibrinolytics were added, as evidenced by no significant change in the percentage of dissociation of tumor cells over time (Fig. 2A,B, black histograms; *P* = ns for 35 min vs. 60 min for both MDA‐MB 231 and A549). A similar trend was observed with the total cell counts (Fig. 2C,D; *P* = ns). Furthermore, the representative images show that there is no increase in the dissociated cells found in the supernatant (Fig. S2).

In the fibrinolytic treatment groups, furthermore, there was a gradual increase in the dissociation percentages after the tPA or TNK is added. A significant increase in dissociation was recorded for both cell lines between 35 and 60 min across all tPA and TNK concentrations (Fig. 2A, MDA‐MB‐231: *P* < 0.0001 for all; Fig. 2B, A549 *P* ≤ 0.0025). Similarly, the same trend of increasing dissociation, measured via supernatant cell counts, was observed with more tumor cells released from the cluster (i) over time following the fibrinolytic addition and (ii) when using higher fibrinolytic dose at 35 min (i.e. 25 min following tPA or TNK addition) (Fig. 2C,D). These same trends are confirmed in the representative images which illustrate the number of dissociated cells in the supernatant over time and with treatment (Fig. S2).

### Platelet–tumor cell cluster dissociation using fibrinolytic drugs

**Fig. 3:**
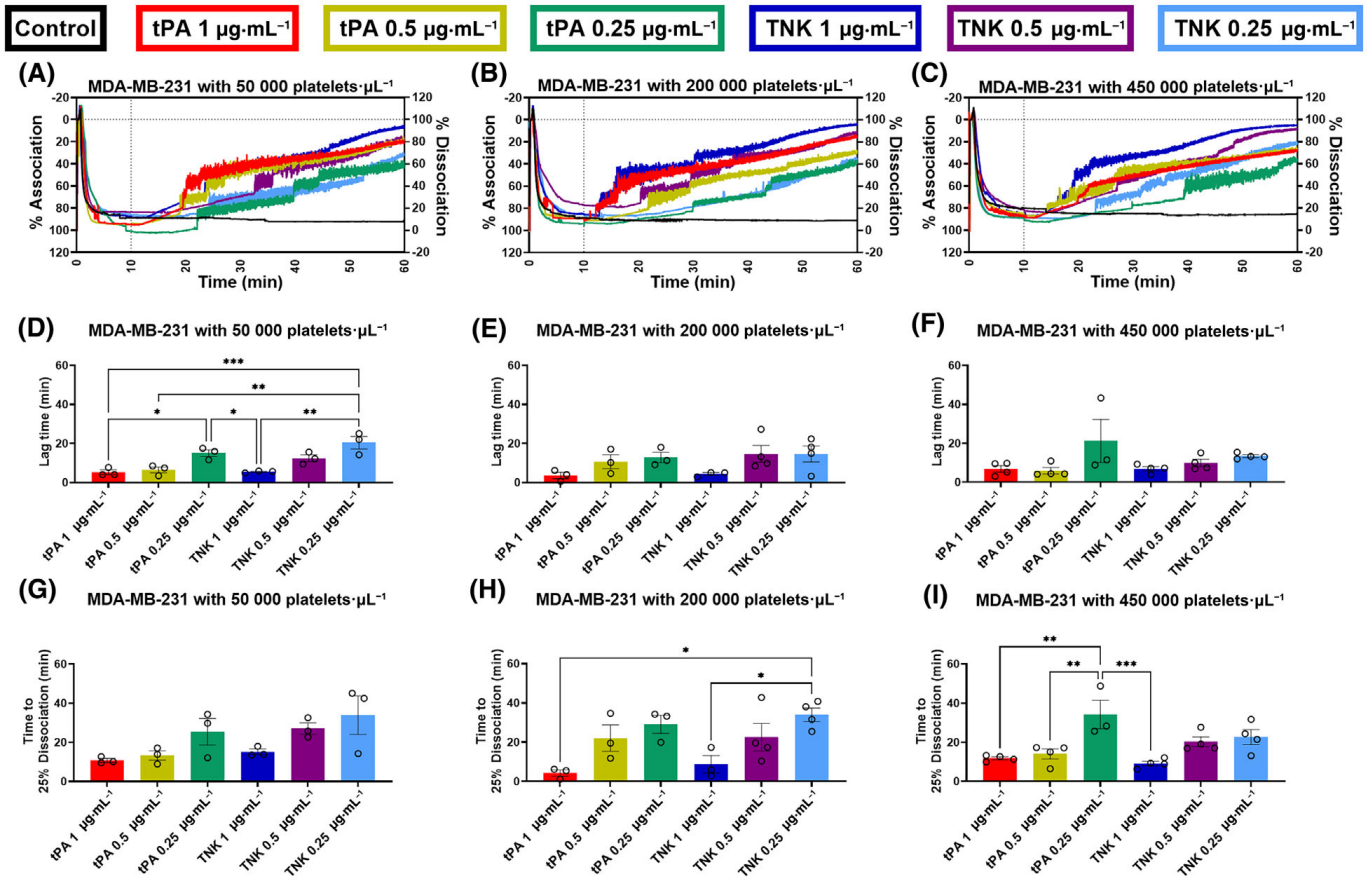
Characterization of light transmission aggregometry (LTA) curves for the association and dissociation of MDA‐MD‐231 cancer cells with low (50 000 platelets·μL^−1^), normal (200 000 platelets·μL^−1^), and high (450 000 platelets·μL^−1^) concentrations of platelets. The LTA curves for the MDA‐MB‐231 tumor cell association, and dissociation in presence of platelets after treatment with different concentrations of tissue plasminogen activator (tPA) and tenecteplase (TNK), were analyzed to produce an average curve (A–C), determine the dissociation lag time (D–F), and measure the time to 25% disassociation (G–I). The tPA and TNK conditions were: control (all platelet concentrations: *n* = 4), 1 μg·mL^−1^ tPA (50 000 platelets·μL^−1^: *n* = 3, 200 000 platelets·μL^−1^: *n* = 3, 450 000 platelets·μL^−1^: *n* = 4), 0.5 μg·mL^−1^ tPA (50 000 platelets·μL^−1^: *n* = 3, 200 000 platelets·μL^−1^: *n* = 3, 450 000 platelets·μL^−1^: *n* = 4), 0.25 μg·mL^−1^ tPA (all platelet concentrations: *n* = 3), 1 μg·mL^−1^ TNK (50 000 platelets·μL^−1^: *n* = 3, 200 000 platelets·μL^−1^: *n* = 3, 450 000 platelets·μL^−1^: *n* = 4), 0.5 μg·mL^−1^ TNK (50 000 platelets·μL^−1^: *n* = 3, 200 000 platelets·μL^−1^: *n* = 4, 450 000 platelets·μL^−1^: *n* = 4), and 0.25 μg·mL^−1^ TNK (50 000 platelets·μL^−1^: *n* = 3, 200 000 platelets·μL^−1^: *n* = 4, 450 000 platelets·μL^−1^: *n* = 4). For each condition, the treatment is added at 10 min as indicated with a dotted line on the average curve (A–C). The lag time (D–F) and time to 25% dissociation (G–I) are measured from the addition of the treatment. To determine the statistical significance of differences between conditions, a one‐way ANOVA analysis with Tukey's multiple comparison test was performed (*P ≤ 0.05, **P ≤ 0.01, ***P ≤ 0.001). For all conditions, the mean and standard error of the mean (SEM) are displayed.

**Fig. 4:**
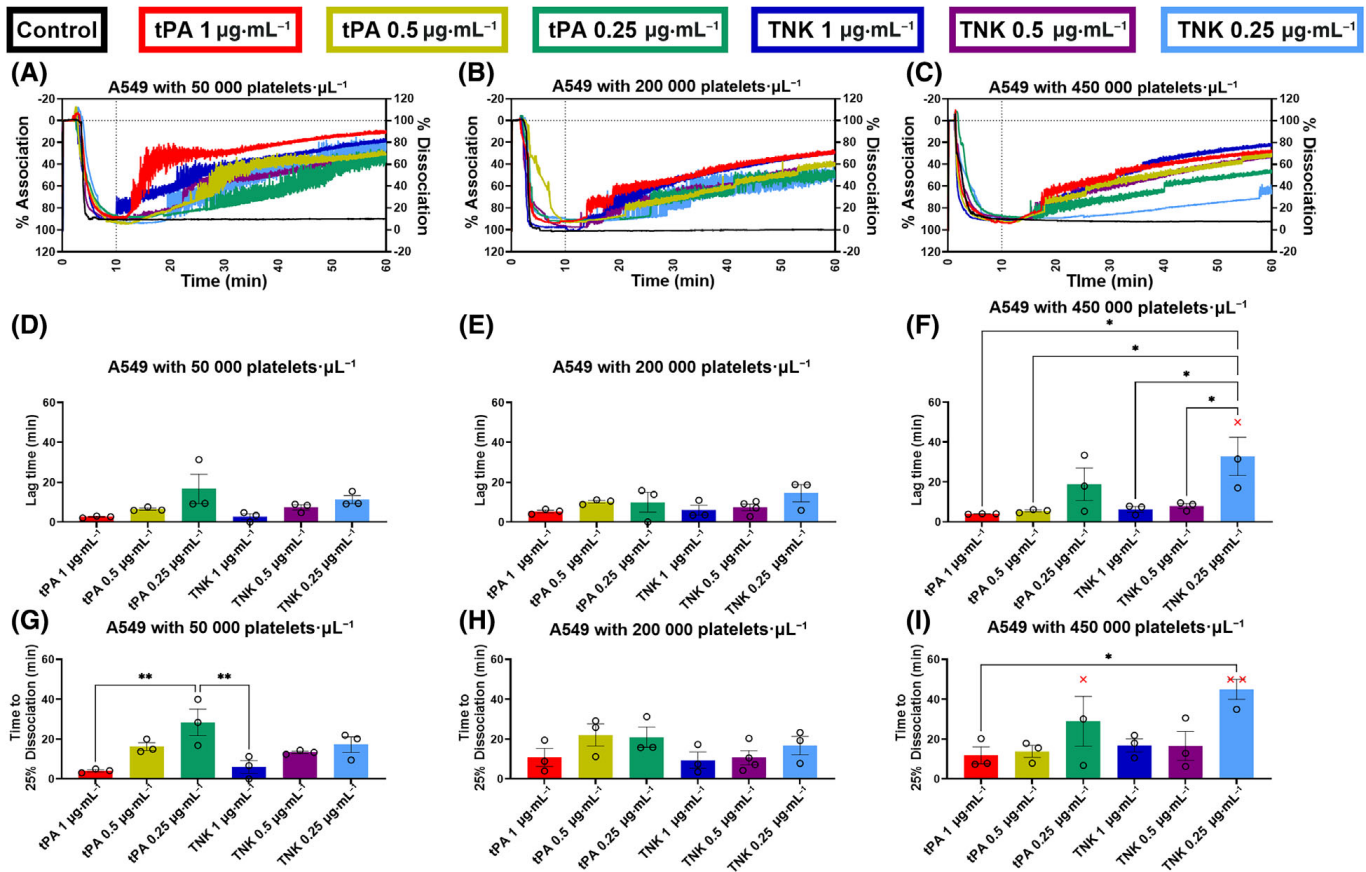
Characterization of light transmission aggregometry (LTA) curves for the association and dissociation of A549 cancer cells with low (50 000 platelets·μL^−1^), normal (200 000 platelets·μL^−1^) and high (450 000 platelets·μL^−1^) concentrations of platelets. The LTA curves for the A549 tumor cell association, and dissociation in presence of platelets after treatment with different concentrations of tissue plasminogen activator (tPA) and tenecteplase (TNK), were analyzed to produce an average curve (A–C), determine the dissociation lag time (D–F), and measure the time to 25% disassociation (G–I). The tPA and TNK conditions were: control (all platelet concentrations: *n* = 3), 1 μg·mL^−1^ tPA (all platelet concentrations: *n* = 3), 0.5 μg·mL^−1^ tPA (all platelet concentrations: *n* = 3), 0.25 μg·mL^−1^ tPA (all platelet concentrations: *n* = 3), 1 μg·mL^−1^ TNK (all platelet concentrations: *n* = 3), 0.5 μg·mL^−1^ TNK (50 000 platelets·μL^−1^: *n* = 3, 200 000 platelets·μL^−1^: *n* = 4, 450 000 platelets·μL^−1^: *n* = 3), and 0.25 μg·mL^−1^ TNK (all platelet concentrations: *n* = 3). For each condition, the treatment is added at 10 min as indicated with a dotted line on the average curve (A–C). The lag time (D–F) and time to 25% dissociation (G–I) are measured from the addition of the treatment. To determine the statistical significance of differences between conditions, a one‐way ANOVA analysis with Tukey's multiple comparison test was performed (*P ≤ 0.05, **P ≤ 0.01). For all conditions, the mean and standard error of the mean (SEM) are displayed.

The association of the MDA‐MB‐231 with platelets at the low, normal, and high concentrations (0–10 min, Fig. 3A–C) was consistent with an average percent association of 90.2 ± 8.2% (*n* = 22) for low platelet concentration, 86.4 ± 10.7% (*n* = 22) for normal platelet concentration and 84.8 ± 8.5% (*n* = 22) for high platelet concentration in MDA‐MB‐231 samples. Similarly, the association of the A549 with platelets at the low, normal, and high concentrations (0–10 min, Fig. 4A–C) was consistent with an average percent association of 90.2 ± 5.4% (*n* = 21) for low platelet concentration, 94.8 ± 6.1% (*n* = 22) for normal platelet concentration and 89.7 ± 6.7% (*n* = 21) for high platelet concentration in A549 samples. Additionally, the control group demonstrated that this association was firm for at least 1 h without a fibrinolytic treatment for all doses of platelets and for both cell lines (Figs 3A–C and 4A–C, black traces). When the fibrinolytic agent was added at 10 min in the other conditions, all of them showed a strong dissociation with some variations in the time before the start of dissociation.

The lag time set at 5% dissociation was significantly greater for the low doses of 0.25 μg·mL^−1^ for both tPA (*P* = 0.0269) and TNK (*P* = 0.0011) compared to the high doses of 1 μg·mL^−1^ with MDA‐MB‐231 cells and low‐dose platelets (Fig. 3D). A similar trend was observed for the time to 25% dissociation with all three platelet concentrations, although non‐significant (Fig. 3G). A significant difference was observed between 0.25 μg·mL^−1^ TNK and 1 μg·mL^−1^ TNK (*P* = 0.0346) for the normal platelet concentration (Fig. 3H). With the high concentration of platelets, the 0.25 μg·mL^−1^ tPA took significantly longer time to reach the 25% dissociation mark as compared to when 0.5 μg·mL^−1^ tPA (*P* = 0.0066) and the 1 μg·mL^−1^ tPA (*P* = 0.0026) was added (Fig. 3I). Similarly, for the A549 cells with high dose of platelets the lag time was significantly greater for the 0.25 μg·mL^−1^ TNK as compared to the 1 μg·mL^−1^ TNK (*P* = 0.0314) and the 0.5 μg·mL^−1^ TNK (*P* = 0.0462) with one replicate not reaching 5% dissociation over 50 min (Fig. 4F, red × symbol). The time to 25% dissociation was significantly higher for the 0.25 μg·mL^−1^ tPA as compared to the 1 μg·mL^−1^ tPA (*P* = 0.0040) for the low platelet conditions (Fig. 4G). Not all replicates composed of clustered A549 cell with the high dose of platelets reached the 25% dissociation over 50 min when the lowest dose of tPA and TNK was administered (Fig. 4I).

### Formation of platelet–tumor cell clusters

**Fig. 5:**
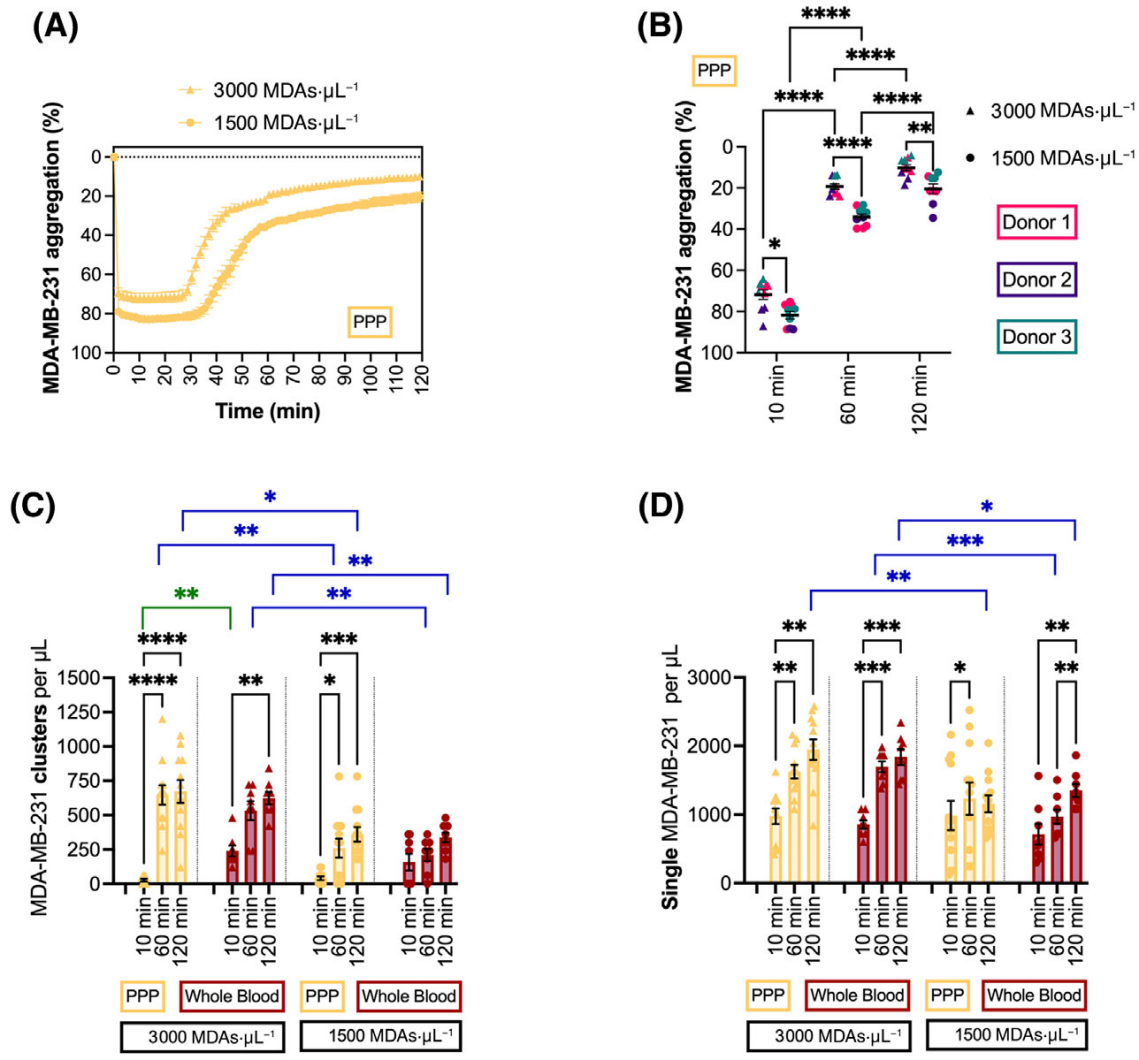
Formation and assessment of platelet–tumor cell clusters by light transmission aggregometry (LTA) and microscopy. The LTA curves for the aggregation of MDA‐MB‐231 cancer cells (MDAs) and platelets into clusters when exposed to calcium chloride (CaCl_2_) supplemented plasma were analyzed to generate (A) an average curve for two cell concentration conditions: 3000 cells·μL^−1^ (*n* = 10) and 1500 cells·μL^−1^ (*n* = 9), (B) determine percent aggregation at 10, 60, and 120 min both cell concentration conditions: (3000 cells·μL^−1^: *n* = 10, 1500 cells·μL^−1^: *n* = 9), (C) determine the number of cancer cell clusters present per μL at 10, 60, and 120 min for two cell concentration conditions in both platelet‐poor plasma (PPP) and whole blood: 3000 cells·μL^−1^ (PPP: *n* = 12; whole blood: *n* = 8) and 1500 cells·μL^−1^ (PPP: *n* = 12; whole blood: *n* = 8), and (D) determine the number of single cells present per μL at 10, 60, and 120 min for two cell concentration conditions: 3000 cells·μL^−1^ (PPP: *n* = 12; whole blood: *n* = 8) and 1500 cells·μL^−1^ (PPP: *n* = 12; whole blood: *n* = 8). A two‐way ANOVA analysis with a Sidak's multiple comparison test was used to determine the effect of cell concentration on platelet–tumor cell cluster aggregation at each time point, while a two‐way ANOVA analysis with a Tukey's multiple comparison test was used to analyze the measured platelet–tumor cell cluster formation over time within each concentration condition (B). Two‐way ANOVA analyses with Tukey's multiple comparison tests were also used to compare the supernatant cancer cell cluster or single cell concentration over time within condition groups (C, D; black *P* values symbols), between samples with different concentration conditions at each time point (C, D; blue *P* value symbols), and between samples in PPP and whole blood for each concentration and time point (C, D; green *P* value symbols) (*P ≤ 0.05, **P ≤ 0.01, ***P ≤ 0.001, ****P ≤ 0.0001). For all conditions, the mean and standard error of the mean (SEM) are displayed.

The formation of platelet–tumor cell clusters was also observed and characterized for MDA‐MB‐231 cell concentrations of 3000 and 1500 cells·μL^−1^ (Fig. 5). The platelets and tumor cells initially formed a large cluster recording a high aggregation: 71.8 ± 7.3% (*n* = 10) for 3000 cells·μL^−1^ and 81.7 ± 5.6% (*n* = 9) for 1500 cells·μL^−1^ at 10 min (Fig. 5A,B). However, these larger clusters are not stable and begin to disaggregate into smaller clusters after the first 25 min (Fig. 5A). The disaggregation started to plateau after 60 min at approximately 19.3 ± 4.2% aggregation for 3000 cells·μL^−1^ and 34.1 ± 4.5% aggregation for 1500 cells·μL^−1^ (Fig. 5A,B). From 60 to 120 min, there was an average of 9.1% and 13.7% further disaggregation for the 3000 cells·μL^−1^ and the 1500 cells·μL^−1^ conditions, respectively.

A similar trend was observed with the concentration of platelet–tumor cell clusters observed in the supernatant of the samples. For the 3000 cells·μL^−1^ and the 1500 cells·μL^−1^ incubated in PPP, there was a significant increase in tumor cell clusters released from 10 to 60 min (Fig. 5C, *P* < 0.0001, and *P* = 0.0203, respectively). Importantly, there was no significant difference between the cluster formed in PPP or whole blood at 60 min or at 120 min (Fig. 5C). Additionally, there is a plateau in the cluster population from 60 to 120 min with no significant change in the cluster count for both PPP or whole blood (Fig. 5C, *P* = ns). The population of single cells in the 3000 and 1500 cell·μL^−1^ conditions incubated in PPP and whole blood also increased from 10 to 60 min in a similar manner (Fig. 5D). Likewise, a plateau in single cell count can also be observed for both cell concentration conditions incubated in PPP and in the 3000 cancer cells·μL^−1^ condition incubated with whole blood (Fig. 5D, *P* = ns).

The supernatant's cluster concentration is significantly higher for the samples formed in PPP with 3000 MDA‐MB‐231/μL concentration compared to the 1500 MDA‐MB‐231/μL concentrations at the 60 min (Fig. 5C, *P* = 0.0042) and 120 min time points (Fig. 5C, *P* = 0. 0247). A similar significant difference is seen between 3000 MDA‐MB‐231/μL and 1500 MDA‐MB‐231/μL concentrations in whole blood at 60 and 120 min (Fig. 5C, *P* ≤ 0.0089). This data is also reflected in the single cell counts in the supernatant which show a significantly higher count for the 3000 MDA‐MB‐231/μL concentration compared to the 1500 MDA‐MB‐231/μL concentration for the PPP samples at 120 min (Fig. 5D, *P* = 0.0026) and for the whole blood samples at both time points (Fig. 5D, *P* ≤ 0.0276). It is also important to note that for all time points and concentrations except the 3000 MDA‐MB‐231/μL at 10 min, there is no significant difference between the samples formed in PPP and those in whole blood in terms of cluster count (*P* = ns) or single cell count (*P* = ns).

## D**i**scussion

Here, we introduce a new method to quantitatively study the association and dissociation of tumor cell clusters leveraging a light transmission aggregometer. The quantitative characterization can be used to investigate the effect of fibrinolytic agents on tumor cell clusters, as well as to monitor cluster preparation for subsequent studies. This method also provides the advantage of observing tumor cell dissociation over time following treatment in a quantitative *in vitro* assay, which could then further aid in designing robust *in vivo* studies.

To develop this assay for tumor cell cluster analysis, we had to adapt standard LTA operation procedures [26]. In brief, we added steps to create an appropriate optical reference, we designed a procedure to initiate consistent tumor cell association, and selected parameters to evaluate tumor cell dissociation. We replaced the plasma reference used in the standard LTA protocol with the supernatant produced after the tumor cells have clustered. This enabled us to accurately reference (in the reference cuvette) the level of turbidity in our samples for 100% aggregation. Another key aspect for this assay was the precise sequence for the addition of the reagents. For example, the CaCl_2_ was added first as it must coat the bottom of the cuvette/stirrer in order to immobilize the cancer cell cluster at the bottom of the well, so as to avoid disturbance with the light path in front of the detector (located mid‐height of the cuvette). Subsequently, a 1 : 1 ratio of tumor cell suspension and plasma must be added immediately before starting the measurement. Finally, to initiate dissociation, we added the fibrinolytic agents at 10 min to allow for stable clustering of tumor cells prior to initiating fibrinolysis. By implementing these modifications, we were able to establish a robust and reproducible protocol for this assay.

With our developed method, we addressed the need for a quantifiable *in vitro* assay for tumor cell dissociation. To demonstrate the value/benefit of this method, we measured and characterized the dissociation of tumor cell clusters with two clinical grade fibrinolytic agents, Alteplase (tPA) and Tenecteplase (TNK). Unlike imaging‐based methods, our approach allows for an unbiased assessment and faster quantifiable characterization of the tumor cell clusters. Assessment parameters can potentially include, however are not restricted to, the lag time to dissociation, time to 25% dissociation, and the dissociation over time (Figs 1 and 2). Using this characterization approach for the MDA‐MB‐231 and A549 cell cluster dissociation, we observed a dose‐dependent trend with the tPA and a significant dose effect with the TNK. These results indicate that our assay can successfully identify key differences in the dissociation curves. For example, the lag time characterization of the dissociation curves illustrates that the time required for lowest dose of TNK to induce dissociation of MDA‐MB‐231 tumor cells is significantly longer than any other condition (Fig. 1C). The dissociation curves for the treated A549 cells also show that the lag time of the 0.25 μg·mL^−1^ TNK treatment is significantly longer than the 1 μg·mL^−1^ dose (Fig. 1D). Our results demonstrate that the measured dissociation curves obtained with this LTA methods could be quantitatively characterized using the above‐mentioned parameters. Furthermore, these characterization parameters were chosen to best analyze and assess the effect of tPA and TNK on MDA‐MB‐231 and A549 cancer cells associated with fibrin. However, alternative parameters could be selected to characterize the dissociation of alternate tumor cell clusters in the presence of various treatments. In addition, we have comprehensively validated our data by enumerating cancer cells released from the cancer cell cluster at various time points (Fig. 2), as well as by imaging these cells in supernatants (Fig. S2).

There was no significant dissociation measured for the clustered MDA‐MB‐231 or A549 cells without a fibrinolytic treatment (Fig. 2A,B), which correlated with a lack of dissociation for the untreated conditions of both cell lines, as observed via the measured number of cells in the supernatant (Fig. 2C,D). Additionally, the correspondence between the cell counts and the measured dissociation with the addition of fibrinolytic drugs illustrates the reliability of the LTA method. Indeed, Pearson correlation analysis indicates very high correlation [27] between cell released in supernatants and fibrinolysis‐triggered cluster dissociation for MDA‐MB‐231 regardless of drugs and time (*r*_35min tPA_ = 0.7442; *r*_60min tPA_ = 0.8071; *r*_35min TNK_ = 0.8191; *r*_60min TNK_ = 0.8899; *P* < 0.0001 for all) (Fig. S3). Regarding A549, the correlation was very high at 60 min and high at 35 min (*r*_35min tPA_ = 0.6709; *r*_60min tPA_ = 0.7730; *r*_35min TNK_ = 0.6241; *r*_60min TNK_ = 0.8812; *P* < 0.0001 for all, except *P*_35min TNK_ = 0.0007) (Fig. S3). Furthermore, there was a significant trend of increase in measured dissociation over time for all fibrinolytic treatment conditions across both cell lines (Fig. 2A,B) that was again confirmed with the supernatant cell counts (Fig. 2C,D). The highly consistent results between the percentage dissociation measured, and the dissociated cell counts obtained indicate that our method is accurately measuring the dissociation of the tumor cells. Interestingly, our data shows similar effect in tumor cell dissociation between tPA and TNK treatment groups (1 and 0.5 μg·mL^−1^), and shows lower fibrinolytic effect of TNK at low dose (0.25 μg·mL^−1^). However, previous work has shown that TNK is a more effective fibrinolytic than tPA [28, 29]. In order verify the fibrinolytic activity of our TNK and tPA treatments, we conducted a delay of clot formation assay (in the absence of cancer cells) in the presence of 1, 0.5, and 0.25 μg·mL^−1^ concentrations of both fibrinolytic agents and thrombin (Fig. S4). Our results indicate that the TNK does maintain a higher fibrinolytic activity compared to tPA. This trend is particularly noticeable with the 0.5 μg·mL^−1^ TNK and tPA treatment, however the trend can also be seen with the more rapid clot lysis with the 0.25 μg·mL^−1^ TNK treatment compared to the corresponding tPA treatment (Fig. S4). Based on these results, there is likely an additional interaction between the fibrinolytic agents and the cancer cells during the tumor cell dissociation to account for the unexpected trends seen in the presence of cancer cells. Exploring the mechanism of this interaction in a future study could provide insight into the dissociation of tumor cell clusters and lead to additional approaches to reduce CTC formation.

In order to account for different possible physiological conditions, we used our LTA method to assess the dissociation of tumor cell clusters in the presence of platelets at three different concentrations to emulate low, normal, and elevated platelet counts. Indeed, high platelet counts are associated with poor prognosis [30] and treatments such as chemotherapy can induce thrombocytopenia [31]. Our method was able to successfully measure cancer cell dissociation with fibrinolytic drugs in the presence of platelets (MDA‐MB‐231: Fig. 3A–C; A549, Fig. 4A–C), despite the optically dense cell suspensions. Furthermore, the lag time and time to 25% dissociation analysis demonstrated a dose‐dependent trend for both the tPA and TNK conditions. For each platelet concentration, the higher concentration of tPA and TNK treatments resulted in lower average lag times and lower average times to reach 25% dissociation of MDA‐MB‐231 (Fig. 3D–I) and A549 (Fig. 4D–I). Further analysis indicates that the addition of platelets to the system did not significantly modulate tumor cell cluster's time to 25% dissociation at the high dose of tPA (1 μg·mL^−1^) and with TNK (all concentrations) for MDA‐MB‐231 (Table S1). Regarding A549‐platelet clusters, their time to 25% dissociation with fibrinolytics was unaffected by the presence of platelets, except for the high platelet concentration, that took more time to dissociate compared to the control without platelets and the lower platelet concentrations (Table S2). However, an increase in time to 25% dissociation was observed for all platelet conditions with MDA‐MB‐231 compared to the non‐platelet groups for the low tPA dose of 0.25 μg·mL^−1^ (Table S1). This increase in time was also observed for the MDA‐MB‐231 with 200 000 platelets·μL^−1^ group when compared to the non‐platelet group when treated with 0.5 μg·mL^−1^ tPA (Table S1). A significant increase in time to 25% dissociation was also observed for the A549 cell clusters with high platelet conditions and treated with the 0.25 μg·mL^−1^ TNK compared to the clusters without platelets, as well as with low and normal platelet conditions treated with the same TNK concentration (Table S2). Thus, the platelet conditions can influence the effectiveness of a fibrinolytic treatment for dispersing tumor cell clusters dependent on the characteristics of the cells. Our LTA method provides one approach for assessing a potential treatment to disperse tumor cell clusters under varying platelet concentrations in a quantitative manner. This would allow for further refinement of dosing for different platelet conditions and for a better understanding of the optimal conditions for a treatment approach. As such, experiments with our LTA method could be used to determine the optimal conditions for each treatment and to potentially develop treatment regimens for specific cancer types.

After using this LTA method to characterize the fibrinolytic dissociation of tumor cell clusters, we next explored the possibility of using this approach to create consistent platelet–tumor cell clusters by mixing platelets with two different concentrations of MDA‐MB‐231 cells in CaCl_2_ supplemented plasma. The formation of these clusters can be measured by the aggregation observed with the LTA apparatus (Fig. 5A) with consistent results between different donors (Fig. 5B). This indicates that the developed method, utilizing a reduced plasma CaCl_2_ concentration with the platelet and cancer cell suspensions, can reliably produce platelet–tumor cell clusters with similar characteristics by forming large aggregates that dissociate into biologically relevant clusters of platelets and tumor cells. The released platelet–tumor cell clusters in the supernatant remain largely stable for a significant period after formation. Indeed, the cluster concentrations remained consistent from 60 to 120 min for all condition groups (Fig. 5C, *P* = ns for all groups between 60 and 120 min) with a high concentration of cancer cell clusters for downstream experiments (e.g. 670 ± 285 clusters·μL^−1^ starting from 3000 MDA‐MB‐231/μL, and 622 ± 128 clusters·μL^−1^ starting from 1500 MDA‐MB‐231/μL at 120 min, Fig. 5C). The single cell concentration for the 1500 cells·μL^−1^ in whole blood condition increased between 60 and 120 min (Fig. 5D, *P* = 0.0068); however, this did not translate in a significant variation of the cluster concentration in this sample (Fig. 5D). Additionally, we observed a concentration effect on the formation of platelet–cancer cell clusters, with higher concentrations producing simultaneously more clusters (Fig. 5C). In addition, between 60 and 120 min, there was no statistical difference in platelet–cancer cell cluster or single cell counts between samples in PPP and in whole blood (Fig. 5C,D, *P* = ns). Therefore, the platelet–tumor cell cluster formation is not significantly altered in whole blood compared to PPP.

**Fig. 6:**
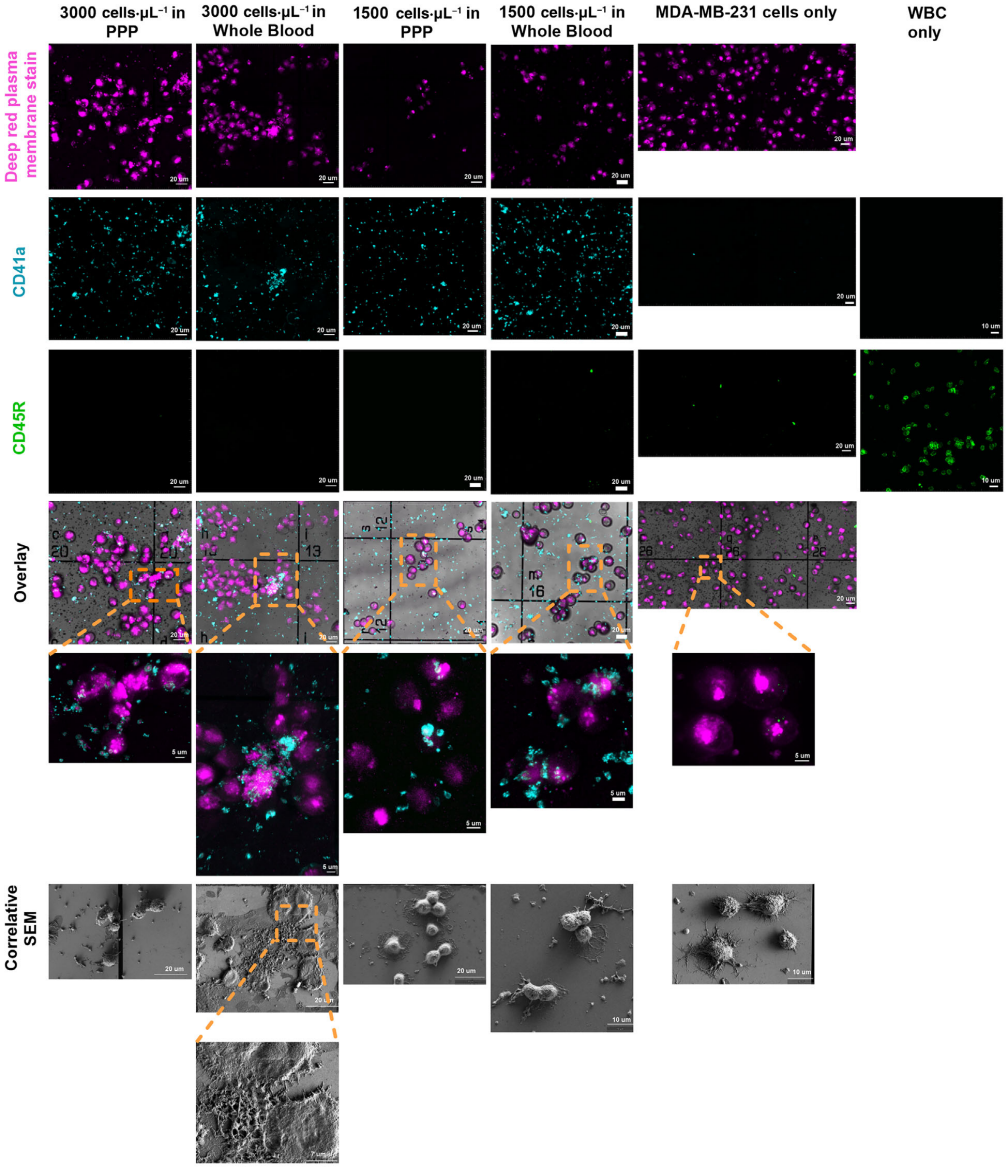
Correlative Light Electron Micrographs (CLEM) for the characterization of the platelet–tumor cell clusters in platelet‐poor plasma (PPP) and whole blood. The MDA‐MB‐231 cancer cells were stained with the deep red plasma membrane stain. The platelets were stained with purified anti‐human CD41 primary antibody and AF488‐conjugated goat anti‐mouse IgG secondary antibody. The white blood cells (WBCs) were stained with purified anti‐mouse/human CD45R/B220 primary antibody and AF546‐conjugated goat anti‐rat IgG secondary antibody (*n* = 2). For the fluorescent images of the WBCs, a 10 μm scale bar was included. For the remaining images examining the CD41a and CD45R fluorescent staining, a 20 μm scale bar was included. The overlay also includes a 20 μm scale bar, and the inset images for the overlay have a 5 μm scale bar. Finally, the correlative scanning electron microscopy images include a 20 μm scale bar except for the MDA‐MB‐231 only and 1500 cells·μL^−1^ in whole blood conditions, which include a 10 μm scale bar, and the inset for the 3000 cells·μL^−1^ in whole blood condition that has a 7 μm scale bar.

We further performed correlative light electron microscopy (CLEM) to study the composition and morphology of the produced platelet–tumor cell clusters (Fig. 6). During the preparation steps for the SEM, 2.5% glutaraldehyde was used for the fixation and HMDS is used to dry the sample overnight. Both glutaraldehyde [32] and HMDS [33] are known to cause shrinkage in cell morphology, which resulted the clusters appearing disjointed under SEM. Some cancer cells were also detached during the multiple washes of the fixation and dehydration process. With confocal microscopy, we observed that the cluster sizes were similar between the PPP and whole blood preparations. The platelets were spread non‐homogeneously throughout the clusters and were visually activated at the surface of the cancer cells (Fig. 6). As such, these activated platelets could contribute to the cluster firmness, which corresponds well to our data showing increased time to 25% dissociation for MDA‐MB‐231 cells clustered with platelets compared to those without platelets when treated with the lowest tPA concentration (Table S1). We did not observe the presence of red blood cells, nor white blood cells (WBCs), trapped in the clusters produced using whole blood, in our experimental setting (Fig. 6). CTC clusters can be associated with WBCs and correlates with poor prognosis in gastric cancer following radical gastrectomy [34] or hepatocellular carcinoma [35]. In these studies, 14.4% [34] and 41.6% [35] of patients presented with WBC‐positive clusters. In their study, Guam *et al*. reported that 6.7% of patients with HER‐2^neg^ metastatic breast cancer had detectable WBC‐positive CTC clusters [36]. Li *et al*. [37] reported that 29.27% of patients with non‐small cell lung cancer present with WBC‐positive CTC clusters. WBC‐associated CTC clusters are not found in all patients and the percentage of patients with WBC‐positive CTC clusters varies widely and often represents a minority of clusters, which could potentially explain the lack of WBC‐positive cancer cell clusters in our study. The absence of these cells, and the similar sizes observed for the clusters formed in PPP and whole blood correlate well with MDA‐MB‐231 cluster and single cell concentration data. These results indicate that the platelet–tumor cell clusters are similar when produced in either PRP or whole blood, which supports the conclusion that our method is able to produce biologically relevant platelet–cancer cell clusters in PRP.

Therefore, such clusters could then be used in downstream *in vitro* studies such as transwell invasion assay and drug testing [38], or in subsequent *in vivo* studies requiring size‐calibrated CTC clusters [39]. Additionally, adjusting the cell concentration allowed for control over the size and appearance of the clusters. Further, the LTA curve correlates well with these differences in cluster size, allowing for monitoring of the cluster formation. Moreover, the LTA monitoring of the cluster formation is useful for determining the time required to form consistent clusters. We expect that the time required for the optimally sized clusters to form could vary depending on the tumor cell lines used and their concentration.

Using our method, care must be taken with operator techniques which include: (i) the need to carefully coat the bottom of the cuvette with CaCl_2_ prior to initiating the tumor cell cluster formation. Indeed, if any CaCl_2_ solution is on the walls of the cuvette, the tumor cell clusters formed could potentially block the optical path of the LTA and induce signal interference. Another potential error in measurement could arise if (ii) there are bubbles present in the sample. In addition, if there is a need to extract samples during the experimental run for imaging or cell enumeration, one must be careful (iii) to avoid interrupting the optical path by obstructing the detector window with a pipette tip. Additionally, there are some technical challenges that could interfere with the measurement, such as the pro‐coagulant effect of cancer cells in plasma [40]. Therefore, it is important to mix tumor cell suspensions with plasma and immediately transfer the sample to a cuvette with CaCl_2_. Delays at this stage will result in increased data variability. Furthermore, (iv) once the sample is added to the cuvette, the baseline for the channel must be set immediately as instructed by the aggregometer manufacturer.

Even though some LTA instruments are equipped with 8 channels, the throughput of the assay is limited by the need to produce appropriate optical references and by the time required for each experimental set. Another limitation is the minimum volume requirement, which may pose a challenge for applications where either the sample volume or cell concentration are restricted.

The technique is not suitable to study *ex vivo* CTC clusters (e.g. collected from a small animal model or patients) as it would require a large number of CTC clusters to get a detectable signal. It is also important to note that there may be other factors for cells that enter the bloodstream as clusters, that are not fully represented in this method. This technique forms the clusters in a plasma‐based solution, which most closely replicates tumor cell clusters aggregating in the blood. This approach emphasizes the fibrin network and platelet interactions important for CTC clusters [9, 10, 11, 12, 13]. However, cells that enter the bloodstream as a cluster unit may have additional factors binding them together that are not fully replicated in this approach. In addition, this method could be used to study the effect of fibrinolytic drugs with concomitant chemotherapeutic drugs. The LTA detection in the ChronoLog instrument is done at 940 nm. Thus, chemotherapeutic drugs and drug delivery systems that absorb at this wavelength will interfere with the measurement.

Additionally, the stability of the platelet–tumor cell clusters produced for downstream applications using this method is dependent on the formation time. Using this approach, the platelets and tumor cells initially form large unstable aggregates that begin to dissociate after 25 min (Fig. 5A), producing smaller platelet–tumor cell clusters with the size and concentration of these clusters varying with the initial tumor cell concentration (Fig. 5C,D). These smaller tumor cell clusters remain largely stable from 60 to 120 min; however, the morphology and stability of these clusters have not been assessed beyond this time frame. As such, the formation of these platelet–tumor cell clusters must be monitored via the LTA measurement according to experimental needs.

Finally, fibrinolytic efficiencies have been assessed and validated using MDA‐MB‐231 and A549, while the platelet–tumor cell cluster assay was developed using MDA‐MB‐231. Further validations with a wider selection of tumor cell lines are needed to determine the versatility of these assays. Refining the experimental parameters such as the cancer cell and CaCl_2_ concentrations and/or platelet count could help to modulate the cluster size as well.

## Conclusion

Our method is able to successfully quantify the association and dissociation of tumor cells *in vitro* using a light transmission aggregometer. We have demonstrated that this method can characterize the dissociation curve of tumor cells and platelet–tumor cell clusters, allowing for the comparison of different fibrinolytic treatments with different cell lines under different platelet concentration conditions. Furthermore, we were able to validate this method by recording the number of cells in the supernatant over time, establishing that it can be used to accurately assess differences in tumor cell association and dissociation *in vitro*. This method could potentially be used in a variety of applications such as assessing the effectiveness of new nanoparticle‐based fibrinolytic agents, identifying key factors in tumor cell association/cluster formation, or refining dosing parameters in advance of *in vivo* trials. Thus, this method provides a valuable new approach to understanding and assessing cancer cell clusters. Finally, we further leveraged our LTA method to produce and monitor the formation of consistent, stable platelet–tumor cell clusters that can be utilized for downstream applications.

## Conflict of interest

ALP has an active material transfer agreement with Genentech for the use of Alteplase and Tenecteplase. Authors declare no conflict of interest.

## Author contributions

Conceptualization: RKK, BS, A‐LP, Experimental design: RKK, BS, A‐LP, Data collection: RKK, BS, Data analysis: RKK, BS, A‐LP, Writing and editing: RKK, BS, A‐LP, Grant: A‐LP.

## Supporting information


**Fig. S1.** Light transmission aggregometry data sets of MDA‐MB‐231 and A549 tumor cell association in calcium chloride (CaCl_2_) supplemented plasma and dissociation with fibrinolytic agents.
**Fig. S2.** Representative images for the validation of light transmission aggregometry (LTA) analysis via cell count microscopy of MDA‐MD‐231 and A549 cancer cells.
**Fig. S3.** Correlation between light transmission aggregometry measured cancer cell dissociation and supernatant cell counts after fibrinolytic treatment.
**Fig. S4.** Comparison of the fibrinolytic effectiveness of tPA and TNK.
**Table S1.** Comparison of time to 25% dissociation for MDA‐MB‐231 cancer cell clusters following fibrinolytic treatment in the absence or presence of platelets.
**Table S2.** Comparison of time to 25% dissociation for A549 cancer cell clusters following fibrinolytic treatment in the absence or presence of plate.

## Data Availability

The data that support the findings of this study are available from the corresponding author upon reasonable request.
