# Supplementary material for: A method to quantitatively characterize the formation and dissociation of tumor cell clusters using light transmission aggregometry
Source: Mol Oncol. 2024 Sep 5;19(1):37–55. doi: 10.1002/1878-0261.13723 (PMC11705735; doi:10.1002/1878-0261.13723)
Supplement: Supplementary file 1 — Fig. S1. Light transmission aggregometry data sets of MDA‐MB‐231 and A549 tumor cell association in calcium chloride (CaCl2) supplemented plasma and dissociation with fibrinolytic agents. Fig. S2. Representative images for the validation of light transmission aggregometry (LTA) analysis via cell count microscopy of MDA‐MD‐231 and A549 cancer cells. Fig. S3. Correlation between light transmission aggregometry measured cancer cell dissociation and supernatant cell counts after fibrinolytic treatment. Fig. S4. Comparison of the fibrinolytic effectiveness of tPA and TNK. Table S1. Comparison of time to 25% dissociation for MDA‐MB‐231 cancer cell clusters following fibrinolytic treatment in the absence or presence of platelets. Table S2. Comparison of time to 25% dissociation for A549 cancer cell clusters following fibrinolytic treatment in the absence or presence of plate. [file MOL2-19-37-s001.zip › Legends.docx]

**Supplementary Figure Legends**

**Figure S1. Light transmission aggregometry data sets of MDA-MB-231 and A549 tumor cell association in calcium chloride (CaCl_2_) supplemented plasma and dissociation with fibrinolytic agents.** The % association (left y-axis) and % dissociation (right y-axis) of cancer cells in the presence of tissue plasminogen activator (tPA) or tenectaplase (TNK). (**A**) MDA-MB-231 raw data, and (**B**) smoothed data. (**C**) A549 raw data, (**D**) smoothed data. A1-A7 are the MDA-MB 231 data sets with control (n = 6), tPA 1 ug/mL (n = 10), tPA 0.5 ug/mL (n = 9), tPA 0.25 ug/mL (n = 6), TNK 1 ug/mL (n = 5), TNK 0.5 ug/mL (n = 7), TNK 0.25 ug/mL (n = 8). B1-B7 are the corresponding MDA-MB-231 smoothed data sets. C1-C7 are the A549 data sets with control (n = 7), tPA 1 ug/mL (n = 6), tPA 0.5 ug/mL (n = 8), tPA 0.25 ug/mL (n = 9), TNK 1 ug/mL (n = 5), TNK 0.5 ug/mL (n = 7), TNK 0.25 ug/mL (n = 7). D1-D7 are the corresponding A549 smoothed data sets.

**Figure S2: Representative images for the** **validation of light transmission aggregometry (LTA) analysis via cell count microscopy of MDA-MD-231 and A549 cancer cells.** The respective (**A**) MDA-MB-231 and (**B**)A549 tumor cell association, and dissociation after treatment with different concentrations of tissue plasminogen activator (tPA) and tenecteplase (TNK), were monitored and 5 uL of supernatant collected at 0, 10, 35, and 60 min time points for imaging. Representative images of the 5uL sample collected for each treatment condition are displayed. A 20 um scale bar is also included for reference.

**Figure S3. Correlation between light transmission aggregometry measured cancer cell dissociation and supernatant cell counts after fibrinolytic treatment.** Pearson correlation plots displaying cell count in supernatant versus % dissociation for MDA-MB-231 treated with (A) tPA for 35 min, (B) TNK for 35 min, (C) tPA for 60 min, (B) TNK for 60 min. A549 treated with (E) tPA for 35 min, (F) TNK for 35 min, (G) tPA for 60 min, (H) TNK for 60 min.

**Figure S4. Comparison of the fibrinolytic effectiveness of tPA and TNK.** The absorbance over time at 405 nm was measured for (A) PPP with thrombin (positive control, n=6), PPP with tPA, no thrombin (negative control, n=8), PPP with TNK, no thrombin (negative control, n=8), and PPP, no thrombin (negative control, n=7). (B) tPA and TNK treated PPP at concentrations of 1 ug/mL (tPA, TNK: n = 8), (C) 0.5 ug/mL (tPA: n = 7, TNK: n = 8), and (D) 0.25 ug/mL (tPA: n = 8, TNK: n = 7). A two-way ANOVA analysis with Tukey’s multiple comparison correction was performed to compare the absorbance change due to clot formation for each tPA or TNK treatment or control group. For all conditions the mean and standard deviation (SD) are displayed.

**Table S1.** Comparison of time to 25% dissociation for MDA-MB-231 cancer cell clusters following fibrinolytic treatment in the absence or presence of platelets.

**Table S2.** Comparison of time to 25% dissociation for A549 cancer cell clusters following fibrinolytic treatment in the absence or presence of plate
