# Supplementary figures and images for: A method to quantitatively characterize the formation and dissociation of tumor cell clusters using light transmission aggregometry
Source: Mol Oncol. 2024 Sep 5;19(1):37–55. doi: 10.1002/1878-0261.13723 (PMC11705735; doi:10.1002/1878-0261.13723)

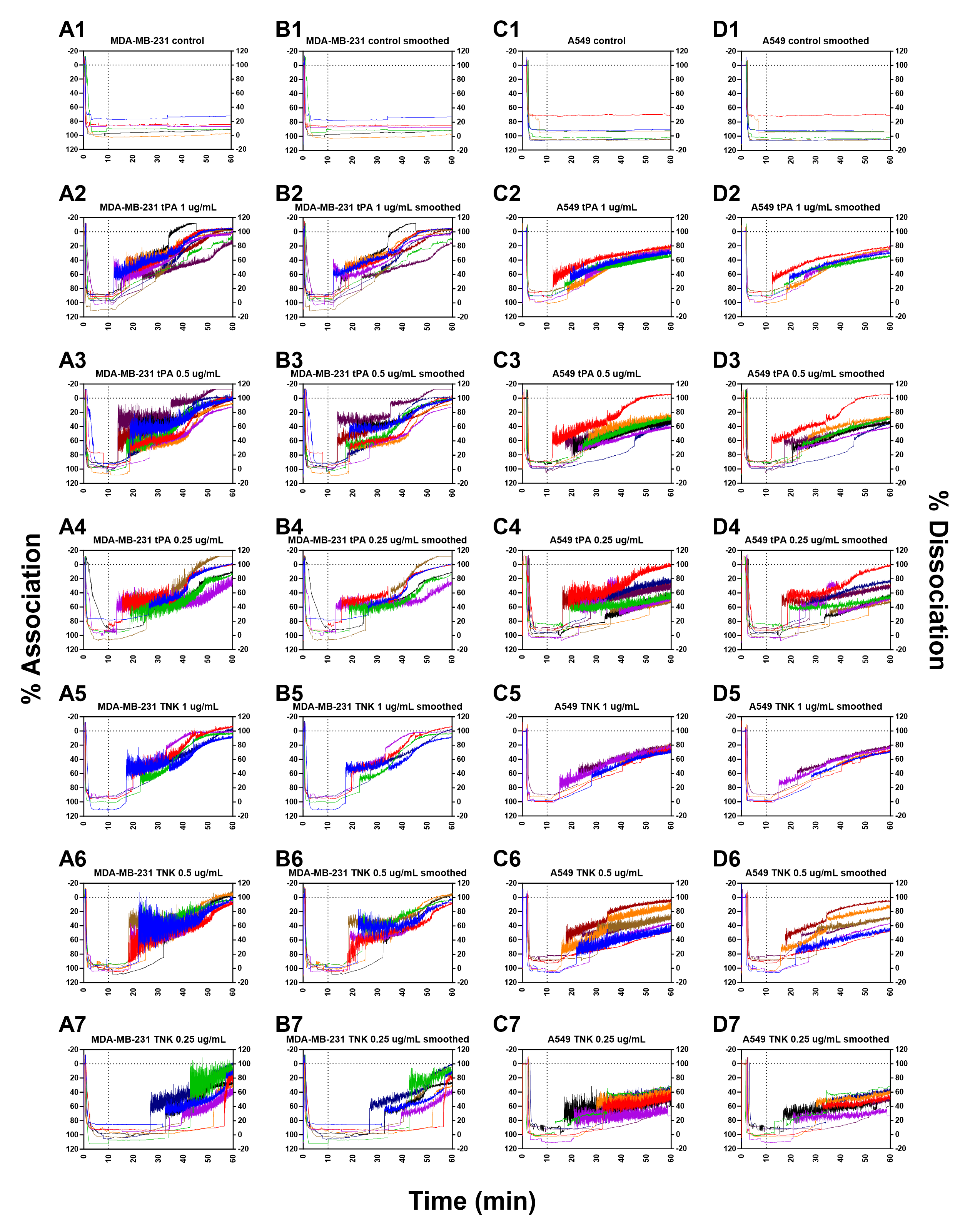

Supplement: Supplementary file 1 — Fig. S1. Light transmission aggregometry data sets of MDA‐MB‐231 and A549 tumor cell association in calcium chloride (CaCl2) supplemented plasma and dissociation with fibrinolytic agents. Fig. S2. Representative images for the validation of light transmission aggregometry (LTA) analysis via cell count microscopy of MDA‐MD‐231 and A549 cancer cells. Fig. S3. Correlation between light transmission aggregometry measured cancer cell dissociation and supernatant cell counts after fibrinolytic treatment. Fig. S4. Comparison of the fibrinolytic effectiveness of tPA and TNK. Table S1. Comparison of time to 25% dissociation for MDA‐MB‐231 cancer cell clusters following fibrinolytic treatment in the absence or presence of platelets. Table S2. Comparison of time to 25% dissociation for A549 cancer cell clusters following fibrinolytic treatment in the absence or presence of plate. [file MOL2-19-37-s001.zip › mol213723-sup-0001-FigureS1.tif]

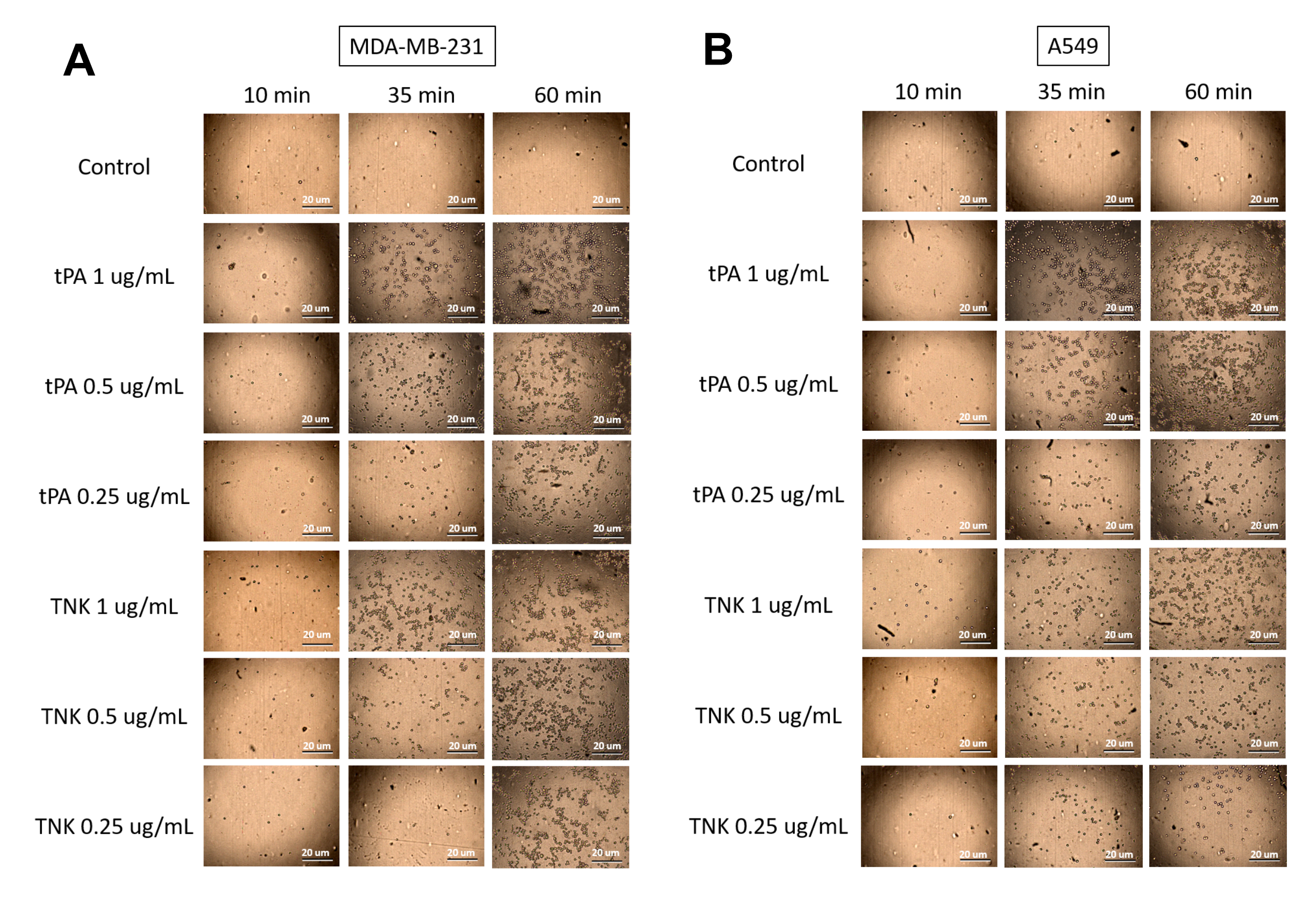

Supplement: Supplementary file 1 — Fig. S1. Light transmission aggregometry data sets of MDA‐MB‐231 and A549 tumor cell association in calcium chloride (CaCl2) supplemented plasma and dissociation with fibrinolytic agents. Fig. S2. Representative images for the validation of light transmission aggregometry (LTA) analysis via cell count microscopy of MDA‐MD‐231 and A549 cancer cells. Fig. S3. Correlation between light transmission aggregometry measured cancer cell dissociation and supernatant cell counts after fibrinolytic treatment. Fig. S4. Comparison of the fibrinolytic effectiveness of tPA and TNK. Table S1. Comparison of time to 25% dissociation for MDA‐MB‐231 cancer cell clusters following fibrinolytic treatment in the absence or presence of platelets. Table S2. Comparison of time to 25% dissociation for A549 cancer cell clusters following fibrinolytic treatment in the absence or presence of plate. [file MOL2-19-37-s001.zip › mol213723-sup-0002-FigureS2.tif]

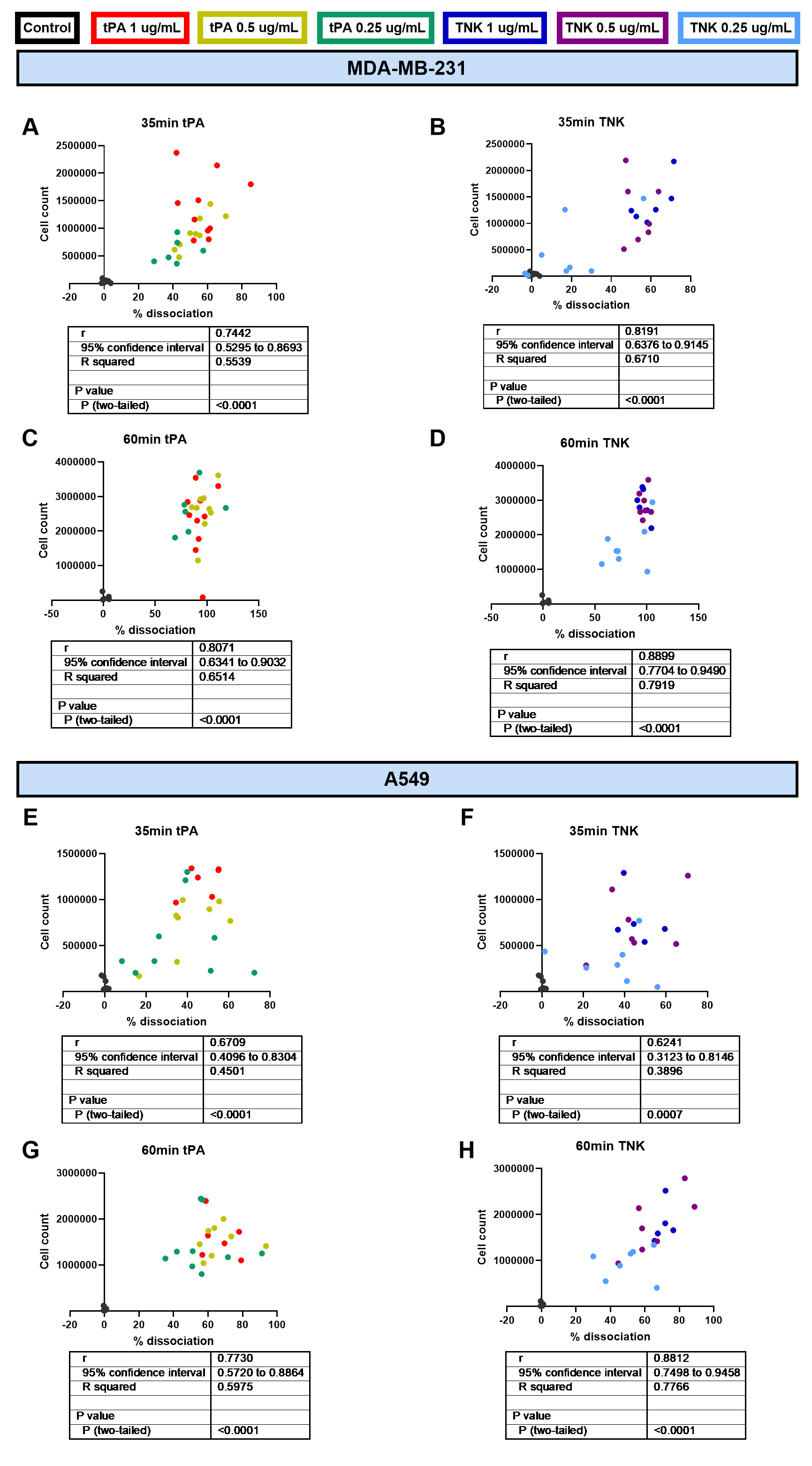

Supplement: Supplementary file 1 — Fig. S1. Light transmission aggregometry data sets of MDA‐MB‐231 and A549 tumor cell association in calcium chloride (CaCl2) supplemented plasma and dissociation with fibrinolytic agents. Fig. S2. Representative images for the validation of light transmission aggregometry (LTA) analysis via cell count microscopy of MDA‐MD‐231 and A549 cancer cells. Fig. S3. Correlation between light transmission aggregometry measured cancer cell dissociation and supernatant cell counts after fibrinolytic treatment. Fig. S4. Comparison of the fibrinolytic effectiveness of tPA and TNK. Table S1. Comparison of time to 25% dissociation for MDA‐MB‐231 cancer cell clusters following fibrinolytic treatment in the absence or presence of platelets. Table S2. Comparison of time to 25% dissociation for A549 cancer cell clusters following fibrinolytic treatment in the absence or presence of plate. [file MOL2-19-37-s001.zip › mol213723-sup-0003-FigureS3.tif]

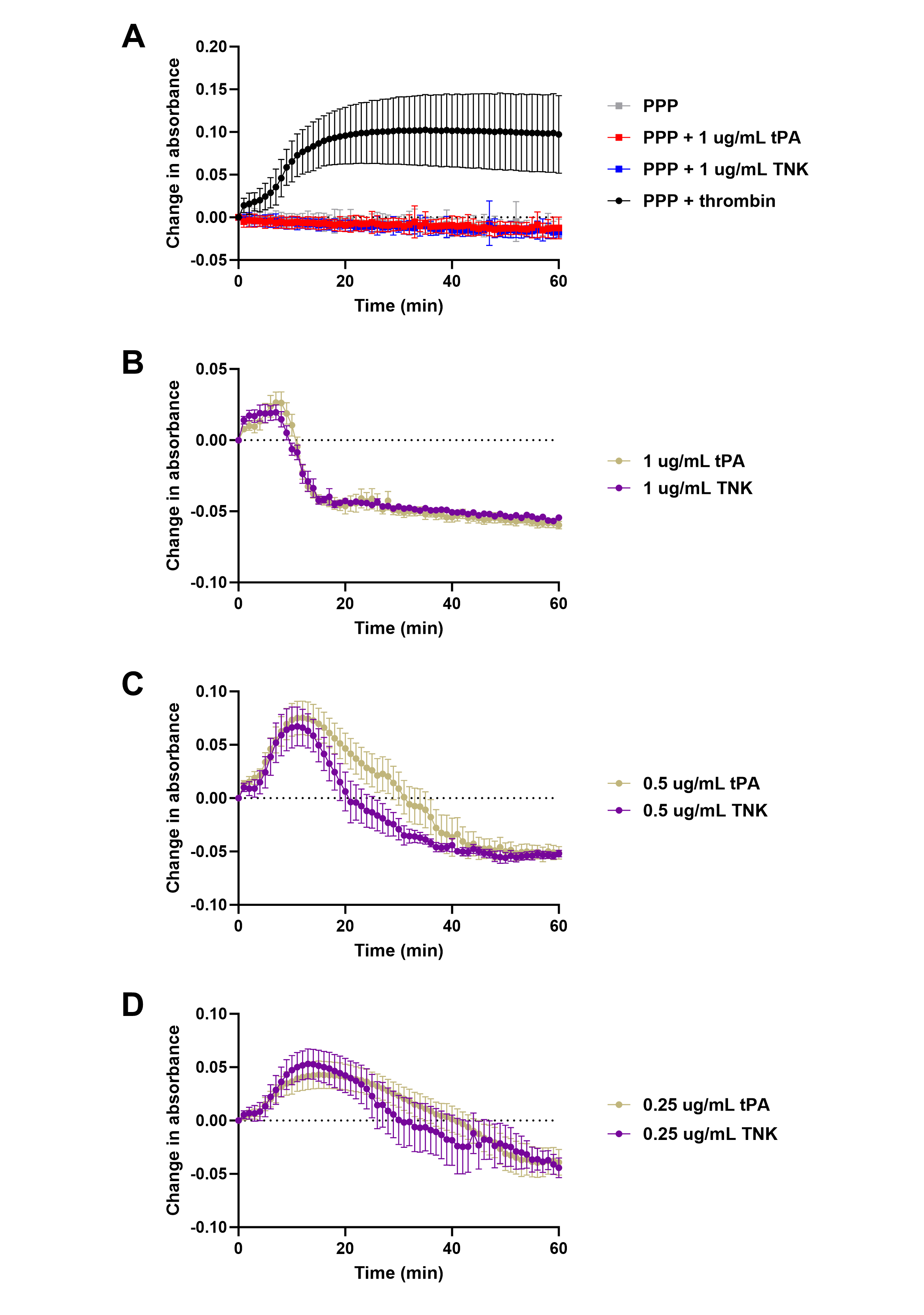

Supplement: Supplementary file 1 — Fig. S1. Light transmission aggregometry data sets of MDA‐MB‐231 and A549 tumor cell association in calcium chloride (CaCl2) supplemented plasma and dissociation with fibrinolytic agents. Fig. S2. Representative images for the validation of light transmission aggregometry (LTA) analysis via cell count microscopy of MDA‐MD‐231 and A549 cancer cells. Fig. S3. Correlation between light transmission aggregometry measured cancer cell dissociation and supernatant cell counts after fibrinolytic treatment. Fig. S4. Comparison of the fibrinolytic effectiveness of tPA and TNK. Table S1. Comparison of time to 25% dissociation for MDA‐MB‐231 cancer cell clusters following fibrinolytic treatment in the absence or presence of platelets. Table S2. Comparison of time to 25% dissociation for A549 cancer cell clusters following fibrinolytic treatment in the absence or presence of plate. [file MOL2-19-37-s001.zip › mol213723-sup-0004-FigureS4.tif]

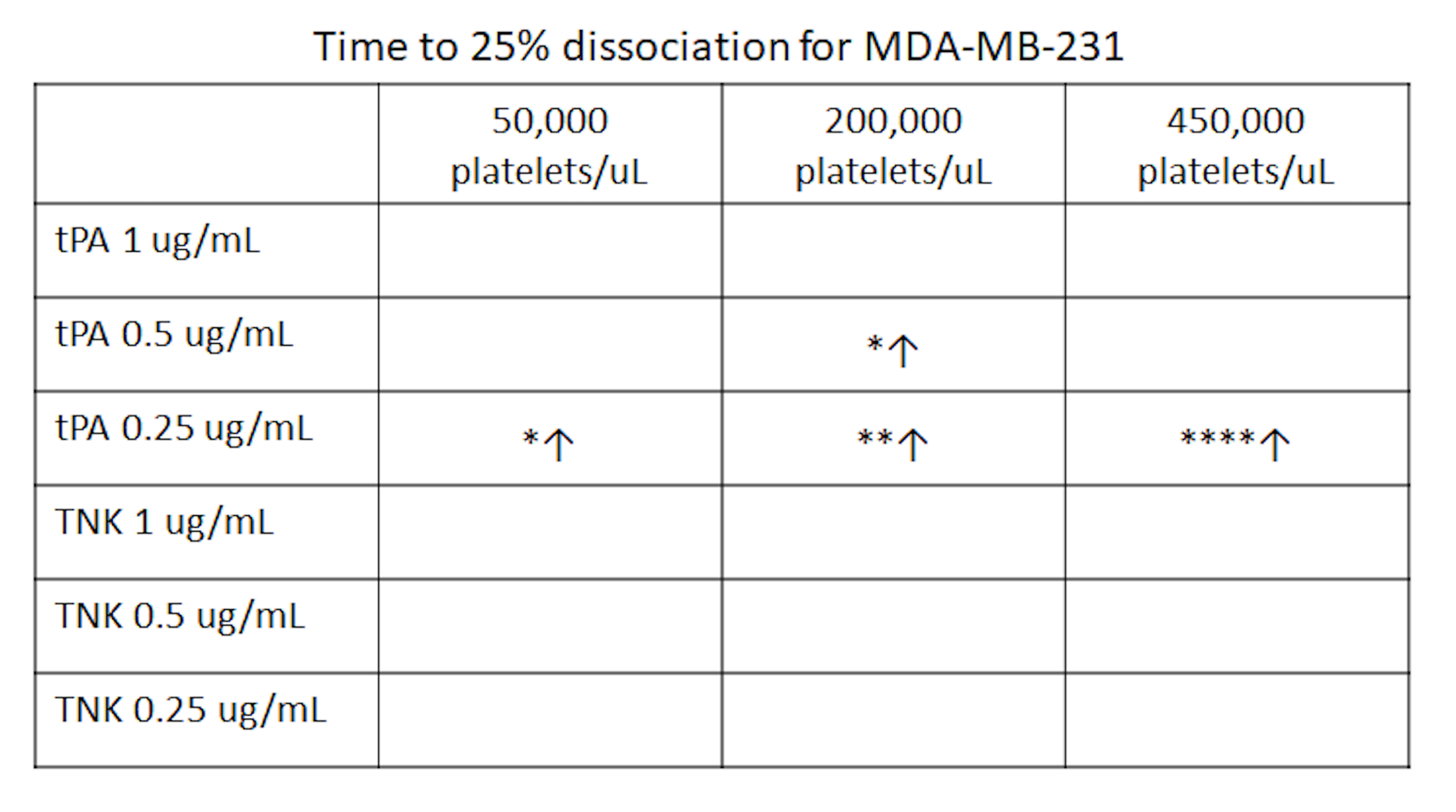

Supplement: Supplementary file 1 — Fig. S1. Light transmission aggregometry data sets of MDA‐MB‐231 and A549 tumor cell association in calcium chloride (CaCl2) supplemented plasma and dissociation with fibrinolytic agents. Fig. S2. Representative images for the validation of light transmission aggregometry (LTA) analysis via cell count microscopy of MDA‐MD‐231 and A549 cancer cells. Fig. S3. Correlation between light transmission aggregometry measured cancer cell dissociation and supernatant cell counts after fibrinolytic treatment. Fig. S4. Comparison of the fibrinolytic effectiveness of tPA and TNK. Table S1. Comparison of time to 25% dissociation for MDA‐MB‐231 cancer cell clusters following fibrinolytic treatment in the absence or presence of platelets. Table S2. Comparison of time to 25% dissociation for A549 cancer cell clusters following fibrinolytic treatment in the absence or presence of plate. [file MOL2-19-37-s001.zip › mol213723-sup-0005-TableS1.tif]

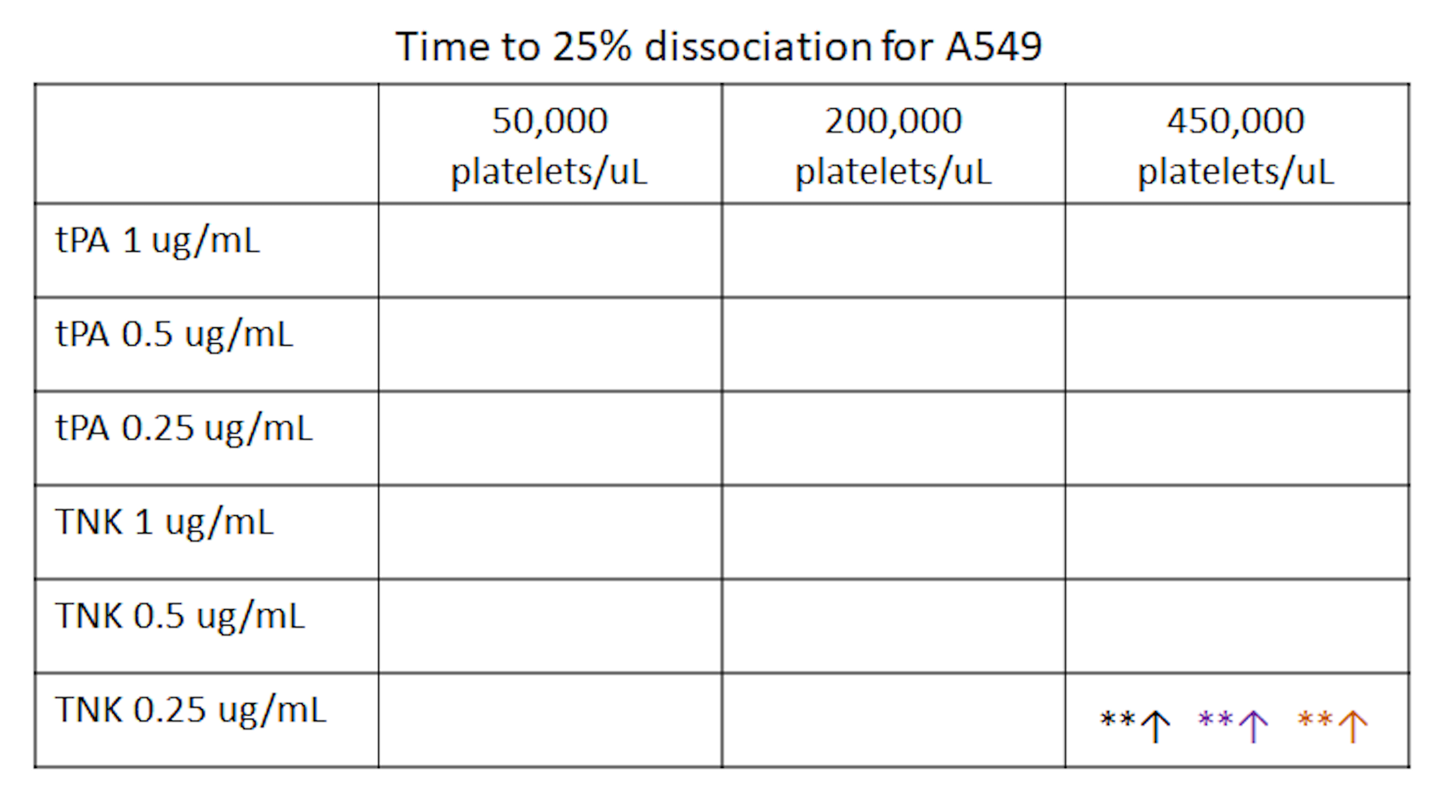

Supplement: Supplementary file 1 — Fig. S1. Light transmission aggregometry data sets of MDA‐MB‐231 and A549 tumor cell association in calcium chloride (CaCl2) supplemented plasma and dissociation with fibrinolytic agents. Fig. S2. Representative images for the validation of light transmission aggregometry (LTA) analysis via cell count microscopy of MDA‐MD‐231 and A549 cancer cells. Fig. S3. Correlation between light transmission aggregometry measured cancer cell dissociation and supernatant cell counts after fibrinolytic treatment. Fig. S4. Comparison of the fibrinolytic effectiveness of tPA and TNK. Table S1. Comparison of time to 25% dissociation for MDA‐MB‐231 cancer cell clusters following fibrinolytic treatment in the absence or presence of platelets. Table S2. Comparison of time to 25% dissociation for A549 cancer cell clusters following fibrinolytic treatment in the absence or presence of plate. [file MOL2-19-37-s001.zip › mol213723-sup-0006-TableS2.tif]
